# NAC-Like Gene *GIBBERELLIN SUPPRESSING FACTOR* Regulates the Gibberellin Metabolic Pathway in Response to Cold and Drought Stresses in *Arabidopsis*

**DOI:** 10.1038/s41598-019-55429-8

**Published:** 2019-12-17

**Authors:** Hong-Ie Chen, Pei-Fang Li, Chang-Hsien Yang

**Affiliations:** 1 Institute of Biotechnology, National Chung Hsing University, Taichung, 40227 Taiwan ROC; 2 Advanced Plant Biotechnology Center, National Chung Hsing University, Taichung, 40227 Taiwan ROC

**Keywords:** Gibberellins, Abiotic

## Abstract

To investigate the functions of *NAC*-like genes, we reported the characterization and functional analysis of one *Arabidopsis NAC*-like gene which showed a novel function in the regulation of gibberellin biosynthesis and named as *GIBBERELLIN SUPPRESSING FACTOR* (*GSF)*. GSF acts as a transcriptional activator and has transactivation capacity based on yeast transcription activity assays. YFP + GSF-TM (lacking a transmembrane domain) fusion proteins accumulated in the nuclei, while the YFP + GSF fusion proteins only accumulated in the ER membrane and were absent from the nuclei. These results revealed that GSF requires processing and release from the ER and transportation into the nucleus to perform its function. The ectopic expression of *GSF-TM* caused a dwarfism phenotype, which was correlated with the upregulation of the gibberellin (GA) deactivation genes GA2-oxidases 2/6 (GA2ox2/6) and the downregulation of the GA biosynthetic genes GA20-oxidases 1–4 (GA20ox1-4). The external application of GA rescued the dwarfism in the 35 S::*GSF-TM* plants, indicating that *GSF* affects GA biosynthesis, rather than the GA signaling pathway. Further analysis indicated that the upregulation of *GA2ox2/6* is a key factor for the *GSF* function to regulate the GA level, since 35 S::*GA20ox1* could not rescue the dwarfism in the 35 S::*GSF-TM* plants. Cold treatment induced the processing of the YFP + GSF fusion proteins from the ER membrane and their entry into the nuclei, which is correlated with the cold-induced upregulation of GA2oxs. In addition, the expression of GA2oxs was induced by drought, and the 35 S::*GSF-TM* plants showed drought tolerance compared to the wild-type plants. Our data suggest a role for *GSF* in response to abiotic stresses, such as cold and drought, by suppressing the biosynthesis of GA in Arabidopsis.

## Introduction

Plant hormone gibberellins (GA) have been hypothesized to play important roles in regulating various developmental processes in plants, such as stem elongation, leaf expansion, seed germination and flower development^1^. Mutants in the gibberellin biosynthetic genes exhibit severe GA-deficiency, such as dwarfism of the inflorescence and compact leaves, in many plant species^2^.

Gibberellins are comprised of tetracyclic diterpene carboxylic acids that require complex biosynthetic steps^3^. To date, many different gibberellins have been identified from plants^4^, but only a small number of gibberellins, such as GA_1_, GA_3_, GA_4_, and GA_7_, function as bioactive hormones. In *Arabidopsis*, the gibberellin biosynthetic genes encode such enzymes as ent-copalyl diphosphate synthase (CPS), ent-kaurene synthase (KS) and ent-kaurene oxidase (KO), which catalyze the early steps of GA biosynthesis. Certain GA metabolic genes, such as GA20-oxidases (GA20oxs) and GA2-oxidases (GA2oxs), are involved in the late steps in the metabolic pathway^5^. GA20oxs participated in the biosynthesis of the major bioactive GAs, including GA_4_, the primary bioactive GA form in *Arabidopsis*. To maintain the dynamic homeostasis of gibberellin for plant growth, the deactivation of GA by GA2oxs is important for the regulation of the concentration of bioactive gibberellin in plants^1,6^.

It has been reported that GA could also play a vital role in response to abiotic stresses in plants^7^. In general, stress may lead to the retardation of growth in plants, and a similar effect is caused by the reduction of GA to adjust its resources to resist the stress^8^. Additional evidence was reported that indicates that GA could respond to abiotic stresses by modulating plant growth via GA biosynthesis or signal transduction^7^. For example, the *GA2oxs* were upregulated to induce the GA deactivation in response to salt and cold stresses in *Arabidopsis*^9–11^. In contrast, the *GA20oxs* were reported to be downregulated in response to cold stress^9,12^. However, the manner in which the mechanisms control GA levels during abiotic stresses still remained to be investigated.

*NAC*-like genes are a group of plant-specific transcription factors that contain a conserved NAC domain (acronym of *NAM* of petunia, *ATAF1*/2 and *CUC2* of *Arabidopsis*) in the N terminal of the proteins^13,14^. The NAC domain consists of approximately 150 amino acids and functions to bind DNA^13^. A typical NAC domain could be divided into five subdomains (A-E). Subdomain A is considered to participate in the formation of the protein dimer. The C and D subdomains were reported to bind to the binding element sequence by a positive charge, while subdomains B and E could confer the functional diversity of NAC^15–17^. The C-terminus of an NAC protein contains a transcriptional regulation region, which has a role to either repress or activate gene expression^18^. NAC transcription factors have been found to be involved in multiple functions that include the development of the shoot apical meristem (SAM)^13,14,19,20^, the manipulation of cell expansion in flower organs^21^, the regulation of lateral root formation^22^, senescence^23,24^, secondary wall biosynthesis in fibers^25^, xylem differentiation^26,27^, anther dehiscence^28,29^ and flower receptivity^30^.

A number of NAC transcription factors contain a transmembrane domain (TM) (designated NTLs) in the C-terminus^31^, which are anchored in the organelle membranes as a dormant form, that have been reported to be closely associated with plant responses to such environmental stresses as UV, drought, heat, cold, osmosis, high salinity, and high-light, and ER stresses, respectively^32–43^. However, the manner in which the mechanisms and factors are regulated by these NTLs in response to environmental stresses has not been thoroughly elucidated to date.

Thus, to further investigate the functions of more *NAC*-like genes is necessary. In this study, we reported the characterization and functional analysis of one *Arabidopsis NAC*-like gene *GIBBERELLIN SUPPRESSING FACTOR* (*GSF). GSF*, also known as RAO2/Arabidopsis NAC domain-containing protein17 (ANAC017), has been reported to be involved in mitochondrial retrograde regulation by acting as a positive regulator of AOX1a^44^. In addition, we identified that its function was associated with GA-related abiotic stress responses. Our results indicated that *GSF* could help the plants respond to cold/drought stresses through the reduction of the GA level by downregulating the GA biosynthetic genes (GA20oxs) and upregulating the GA deactivation genes (GA2oxs) in the GA metabolic pathway. Thus, our study demonstrated a linkage between the *NAC*-like gene and GA in the regulation of the response of plants to abiotic stresses.

## Results

### Isolation of *GSF* cDNA from *Arabidopsis thaliana*

One member of an *Arabidopsis NAC-like* gene, *GIBBERELLIN SUPPRESSING FACTOR* (*GSF)* (At1g34190), was cloned and analyzed. *GSF* contains three introns and four exons (Fig. S1A) and encodes a protein of 557 amino acids (Fig. S1B). The GSF protein contains a putative conserved NAC domain that consists of five subdomains (A-E) (Fig. S1B) and has been identified in the N-terminal ends of most NAC-like proteins^14,45^. In addition, one transmembrane motif is identified in the C-terminal end of the GSF protein^31^ (Fig. S1B). The GSF protein showed 82% similarity and 72% identity to the most closely related NAC-like protein, At1g34180 (Fig. S1B).

### Detection of the expression for *GSF* by the analysis of GSF::*GUS* transgenic *Arabidopsis*

**Figure 1:**
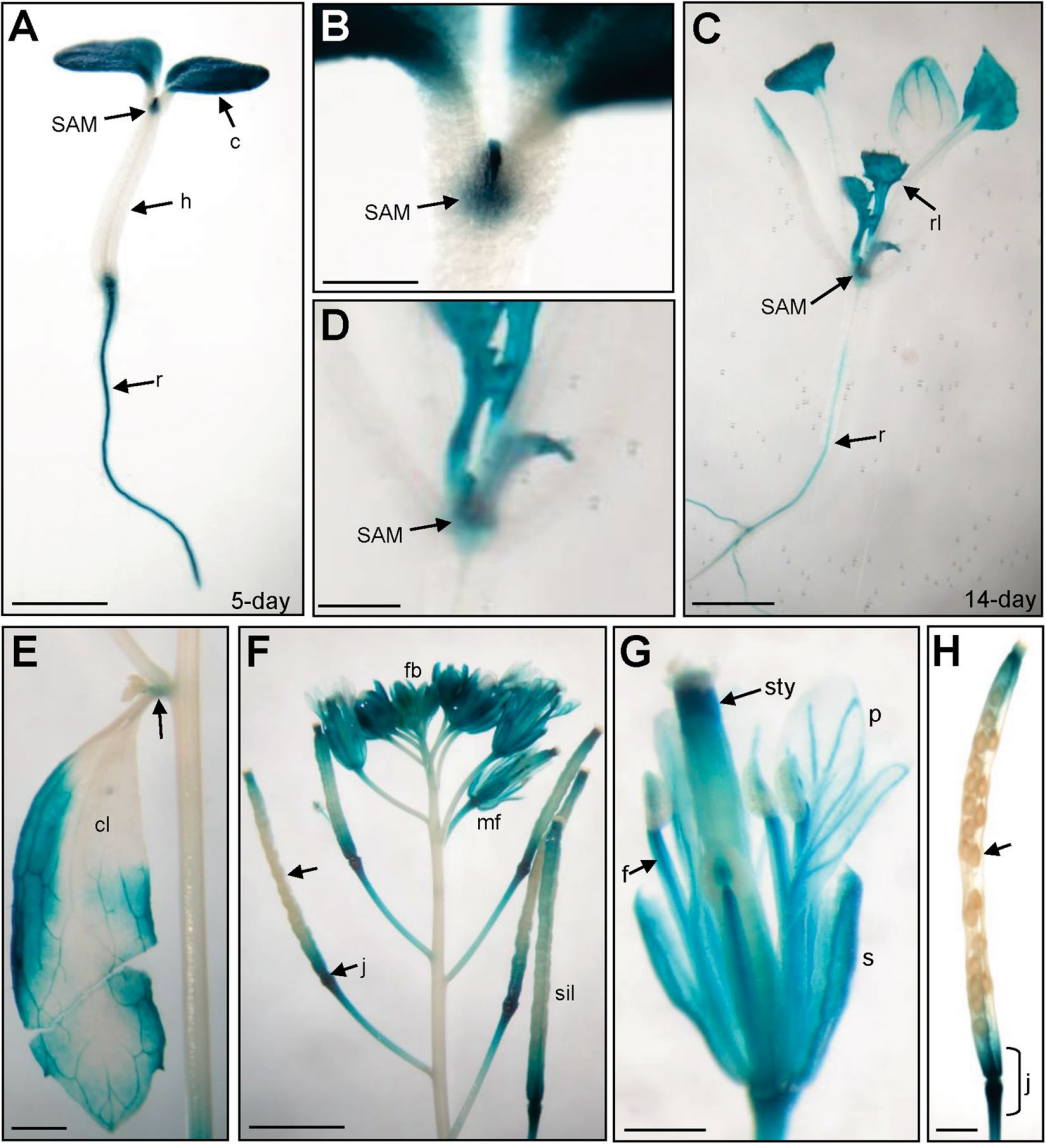
GUS staining patterns in GSF::*GUS* transgenic *Arabidopsis*. (**A**) GUS activity was specifically detected in the cotyledon (c), shoot apex meristem (SAM) and roots (r) of a 5-day-old seedling. GUS was absent in the hypocotyl (h). Bar = 3 mm. (**B**) Close-up of the shoot apex meristem (SAM) region from **(A)**. Bar = 0.5 mm. (**C**) GUS activity was specifically detected in the shoot apex meristem (SAM), rosette leaves (rl) and roots (r) of 14-day-old seedlings. Bar = 3 mm. (**D**) Close-up of the shoot apex meristem (SAM) region from (**C**). Bar = 1 mm. (**E**) GUS activity was detected in the cauline leaf (cl) and the node of inflorescence (arrow) in the inflorescence of a mature plant. Bar = 1 mm. (**F**) In a mature inflorescence, GUS activity was detected in the flower organs in both flower buds (fb) and mature flowers (mf). In siliques, GUS was strongly detected in the junction region (j) of the silique and pedicel and was absent from the developing seeds (arrow). Bar = 25 mm. (**G**) In a mature flower from **(F)**, GUS activity was detected in the sepal (s), stamen filaments (f) and style of the carpel (sty) and weakly detected in the petals (p). Bar = 5 mm. (**H**) In a mature silique from (**F**), GUS was strongly detected in the junction region of the silique and pedicel (j) and was absent in the developing seeds (arrow). Bar = 0.5 mm.

To explore the expression pattern of the *GSF* gene, a construct containing a 2-kb promoter region of *GSF* fused with GUS reporter (GSF::*GUS*) was constructed and transformed into *Arabidopsis*. In the GSF::*GUS* transgenic seedlings, GUS was primarily detected in the root, cotyledon and shoot apex meristem and was absent in the hypocotyl of 5-day-old (Fig. 1A,B) and 14-day-old (Fig. 1C,D) seedlings. In addition, GUS activity was detected in the rosette leaves that had emerged in 14-day-old seedlings (Fig. 1C,D). In mature plants, GUS activity was detected in the rosette and cauline leaves and the node of inflorescence (Fig. 1E). During flower development, GUS activity was primarily detected in the sepal, stamen filaments and style of carpel and weakly detected in the petals in both young and mature flowers (Fig. 1F,G). During silique development, GUS was strongly detected in the junction region of the silique and pedicel and was absent in the developing seeds (Fig. 1F,H). The pattern of GUS expression detected in this study was consistent with the *Arabidopsis* eFP browser data^46,47^ (http://www.bar.utoronto.ca/efp/cgi-bin/efpWeb.cgi).

### GSF acts as a transcriptional activator

**Figure 2:**
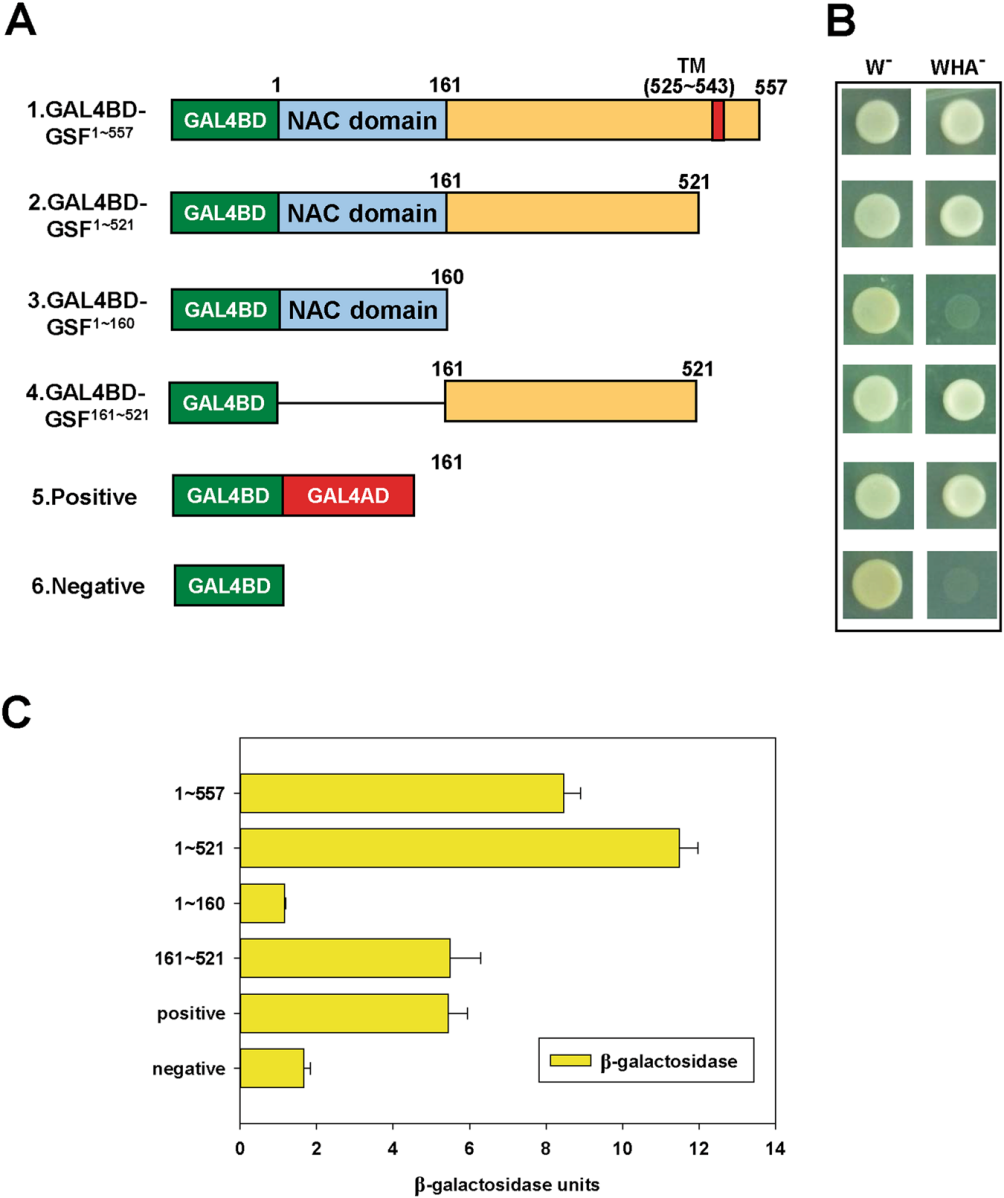
Assay of transcriptional activation for GSF. (**A**) Various forms of GSF, full-length (GSF^1~557^, positions 1–557), truncated transmembrane (GSF^1–521^, positions 1–521), truncated C terminal half (GSF^1–160^, positions 1–160) and truncated N terminal half (GSF^161–521^, positions 161–521), fused with Gal4 DNA-binding domain (GAL4BD) to form four different constructs (GAL4BD-GSF^1~557^, GAL4BD-GSF^1~521^, GAL4BD-GSF^1~160^ and GAL4BD-GSF^161~521^). GAL4BD-GAL4AD is a positive control, while GAL4BD only served as a negative control. (**B**) The six constructs from (**A**) were introduced into the yeast strain AH109 and grown on SD without tryptophan (W^−^) or lacking tryptophan, histidine and adenine (WHA^−^). (**C**) Quantification of the β-galactosidase activity for six different transformants in yeast from (**B**).

To examine whether GSF can act as a transcriptional activator, yeast transcription activity assays were performed. Different constructs containing full-length (GSF1~557, positions 1–557), truncated transmembrane (GSF^1–521^, positions 1–521), truncated C terminal half (GSF^1–160^, positions 1–160) or truncated N terminal half (GSF^161–521^ positions 161–521) forms of GSF (Fig. 2A) fused with the GAL4 DNA-binding domain (GAL4BD) were generated. Two constructs, the GAL4BD–GAL4 activation domain (GAL4AD) and GAL4BD, were constructed as positive and negative controls, respectively (Fig. 2A).

All of the constructs were transformed into the yeast strain AH109 that contains several reporter genes (*lacZ*, *His3*, *MEL1* and *ADE2*) and grew equally well on plates with histidine and adenine (W^−^) (Fig. 2B, left). Comparing the growth of the yeast cells on tryptophan-, histidine- and adenine-minus plates (WHA^−^) (Fig. 2B, right), yeast cells harboring the fusion proteins GAL4BD-GSF^1~557^, GAL4BD-GSF^1~521^ and GAL4BD-GSF^161~521^ grew as well as the positive controls GAL4BD-GAL4AD (Fig. 2B, right). In contrast, yeast cells that harbored the fusion protein GAL4BD-GSF^1~160^ did not grow as well on the WHA^-^ plates as the negative controls GAL4BD (Fig. 2B, right). Further analysis indicated that yeasts harboring GAL4BD-GSF^1~557^, GAL4BD-GSF^1~521^ and GAL4BD-GSF^161~521^ have similar or even higher β–galactosidase activity with the positive controls GAL4BD-GAL4AD (Fig. 2C). Similar low β–galactosidase activity was observed in the yeasts containing GAL4BD-GSF^1~160^ or the negative controls GAL4BD (Fig. 2C). These results indicated that the C-terminus of GSF could activate downstream genes in plants^41^.

### GSF truncated with a transmembrane motif could be released from the ER and enter into the nucleus

**Figure 3:**
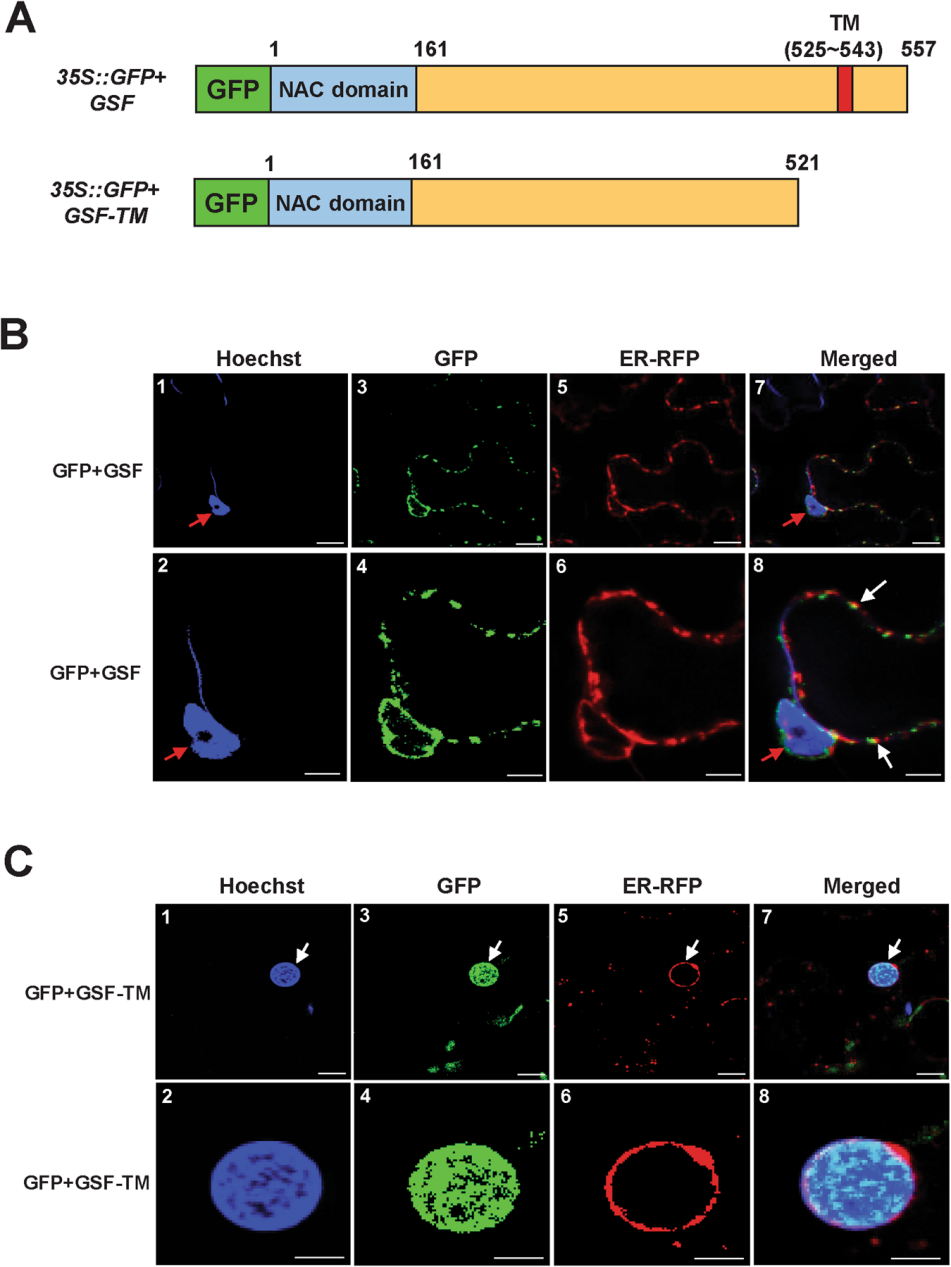
Transient expression of GFP + GSF, GFP + GSF-TM and ER + RFP in tobacco cells. (**A**) Schematic diagram for the GFP + GSF and GFP + GSF-TM constructs transiently expressed in tobacco leaves. (**B**) Agrobacterium-mediated transient expression of GFP + GSF and ER + RFP in the epidermal cells of *N. benthamiana*. The GFP + GSF fusion protein (−3, −4) accumulated in organelle-like structures that were highly similar to the ER where the RFP was localized (−5, −6). Blue DNA-staining (Hoechst) indicated the nuclei (red arrow in −1, −2, −7 and −8). A merged fluorescence image of (−1, −3, −5) in (−7); (−2, −4, −6) in (−8) showing the similar localization of GFP + GSF and ER + RFP (white arrows in −8). (−2, −4, −6 and −8) are close-up images from (−1, −3, −5 and −7), respectively. Scale bars: 10 μm in (−1, −3, −5, −7) and 5 μm in (−2, −4, −6, −8). (**C**) Agrobacterium-mediated transient expression of GFP + GSF-TM and ER + RFP in the epidermal cells of *N. benthamiana*. The GFP + GSF-TM fusion protein (−3, −4) that accumulated in the nucleus (white arrow) was highly similar to the blue DNA-staining (Hoechst) (−1, −2) and different from where ER + RFP was localized (−5, −6). A merged fluorescence image of (−1, −3, −5) in (−7); (−2, −4, −6) in (−8) showing the similar localization of GFP + GSF-TM and blue DNA-staining (Hoechst) (white arrows in −7). (−2, −4, −6 and −8) are close-up images from (−1, −3, −5 and −7), respectively. Scale bars: 10 μm in (−1, −3, −5, −7) and 5 μm in (−2, −4, −6, −8).

It has been reported that a group of NAC transcription factors with a transmembrane motif (designated NTLs) are translocated into the nucleus from the ER (endoplasmic reticulum) to regulate the expression of downstream genes that were activated by a proteolytic cleavage^31^. Since GSF contains a predicted transmembrane motif in the C-terminus that identifies them as NTL proteins^31^ (Figs. 3A, S1B), it is possible that GSF could be triggered and released from the ER and translocated to the nucleus to regulate the expression of the downstream genes. To examine this hypothesis, we transformed GFP fused with the full length GSF (35 S::*GFP* + *GSF*) or the GSF lacking its transmembrane motif (35 S::*GFP* + *GSF-TM*) and *ER-RFP* (as an ER marker) into tobacco leaves, and their fluorescence images were analyzed. The results showed that the fluorescence images of the GFP + GSF fusion proteins matched the RFP fluorescence images of ER-RFP in the ER membrane and were absent from the nuclei (Fig. 3B). In contrast, the GFP fluorescence images of the GFP + GSF-TM fusion proteins only accumulated in the nuclei of the cells, while they overlapped with the blue fluorescence of Hoechst (a dye for DNA -staining) and were absent in the ER membrane (Fig. 3C). These results indicated that GSF was localized to the ER and will be translocated into the nucleus once the transmembrane motif was deleted. This result is in agreement with the previous report^44^, which reported that when the N-terminal red fluorescent protein (RFP) and the C-terminal green fluorescent protein (GFP) are simultaneously tagged on the ANAC017 (GSF) protein, the N-terminal RFP can migrate to the nucleus whereas C-terminal GFP remains in the ER.

### Ectopic expression of *GSF-TM* causes dwarfism of the plants

To explore the function of the *GSF* gene, the full length cDNA and the cDNA lacking the transmembrane motif region of the *GSF* gene, driven by the cauliflower mosaic virus (CaMV) 35S promoter (35S::*GSF* and 35S::*GSF-TM*), were transformed into Arabidopsis for functional analysis.

**Figure 4:**
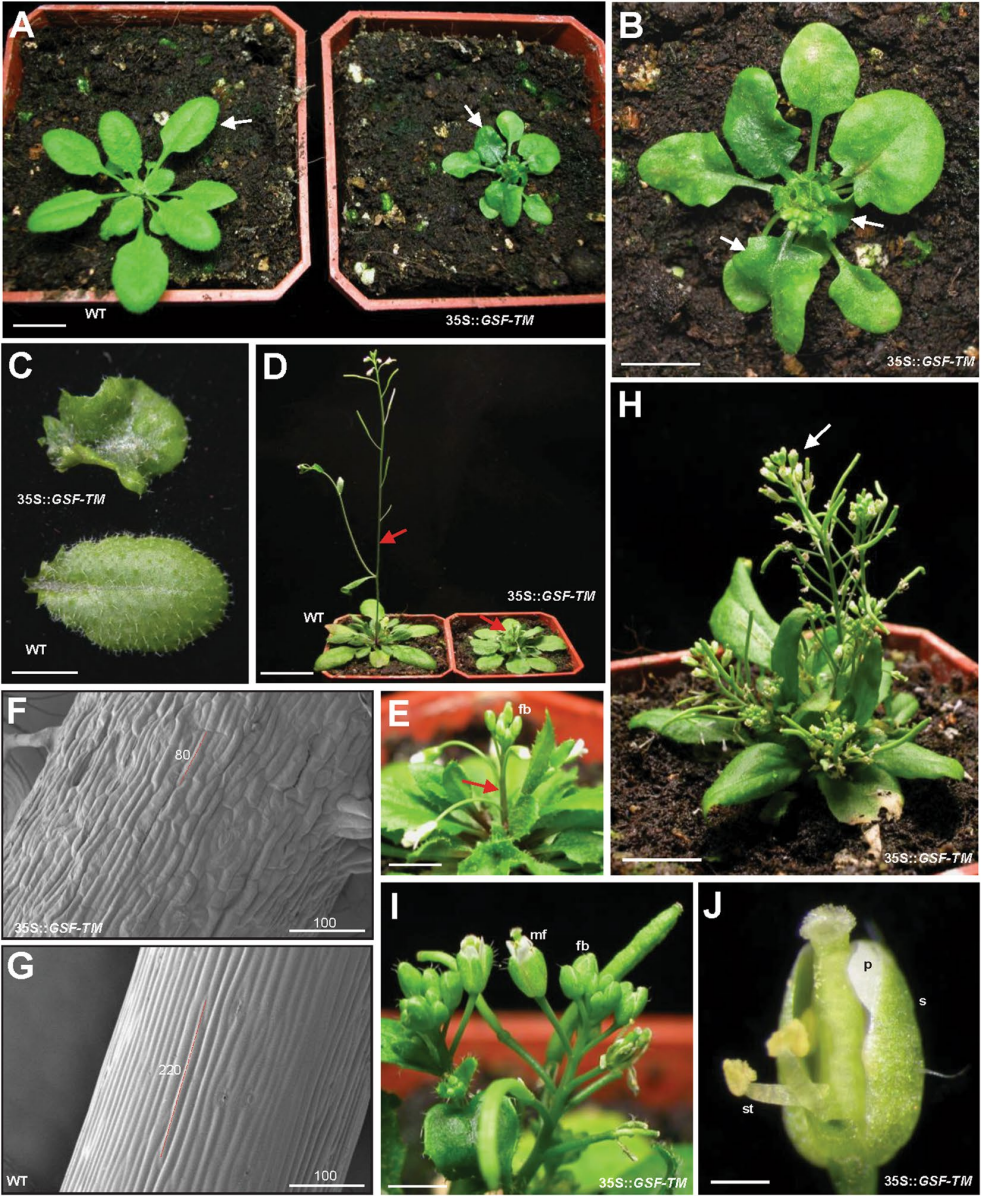
Phenotypic analysis of the 35S::*GSF-TM Arabidopsis* plants. (**A**) A 28-day-old 35S::*GSF-TM* plant (right) showed a severe dwarfism phenotype with small compact and curled leaves (arrow), while wild-type plants (WT, left) produced normal rosette leaves (arrow). Bar = 10 mm. (**B**) Close-up of the 35S::*GSF-TM* plant from **(A)**. Bar = 5 mm. (**C**) Leaf from a severe 35S::*GSF-TM* transgenic plant (top) that showed a compact and curled phenotype. Leaf from a wild-type plant (WT, bottom) with a normal round shape. Bar = 5 mm. (**D**) A 42-day-old 35S::*GSF-TM* plant (right) produced a short inflorescence and internode (arrow), while wild-type plants (WT, left) produced a long inflorescence with normal internode elongation (arrow). Bar = 20 mm. (**E**) Close-up of the inflorescence (arrow) with the floral buds (fb) of a 35S::*GSF-TM* plant from (**D**). Bar = 2 mm. (**F**) Close-up of the epidermal cells (approximately 80 *µ*m in length) in the inflorescence of a 35S::*GSF-TM* plant from **(D)**. Bar = 100 µm. (**G**) Close-up of the epidermal cells (approximately 220 *µ*m in length) in the inflorescence of a wild-type (WT) plant from **(D)**. Bar = 100 µm. (**H**) A 56-day-old 35S::*GSF-TM* plant produced a short inflorescence, internode and compacted flower clusters (arrow). Bar = 10 mm. (**I**) Close-up of the flower buds (fb) and mature flowers (mf) with short flower organs in the inflorescence of a 35S::*GSF-TM* plant from **(H)**. Bar = 2 mm. (**J**) Close-up of a mature flower with short sepal (s), petal (p) and stamen (st) of a 35S::*GSF-TM* plant from **(I)**. Bar = 0.5 mm.

When the 35S::*GSF* Arabidopsis plants were analyzed, they were phenotypically indistinguishable from the wild-type plants in both vegetative and reproductive development. This result revealed that GSF could require processing and release from the ER to perform its function. Interestingly, 35S*::GSF-TM* transgenic *Arabidopsis* plants showed similar abnormal phenotypes, which were significantly different from those of the wild-type plants. These 35S*::GSF-TM Arabidopsis* plants exhibited phenotypic alterations, such as compact and curled rosettes leaves (Fig. 4A–C), short inflorescence internodes and severe dwarfism (Fig. 4D,E). The epidermal cells of the inflorescence in the 35S::*GSF-TM* transgenic plants (80 by 10 micrometers) (Fig. 4F) were significantly shorter than those in the wild type inflorescence (220 by 10 micrometers) (Fig. 4G). During the reproductive stage, flowers with short sepals and petals were observed in the compacted inflorescence of the 35S::*GSF-TM* plants (Fig. 4H–J). These results supported the hypothesis that GSF-TM is the functional form of GSF that regulates plant growth and development. Interestingly, a similar dwarfism phenotype was also observed in the 35S*::GSF-TM* + *VP16* transgenic Arabidopsis (Fig. S2) in which *GSF-TM* was fused to the activation domain VP16-AD^48,49^. This result confirmed the conclusion from the yeast transcription activity assays (Fig. 2), which indicated that GSF acts as a transcriptional activator in plants.

### Gibberellin (GA) biosynthesis genes were downregulated and gibberellin deactivation genes were upregulated in 35S::*GSF-TM* transgenic *Arabidopsis*

**Figure 5:**
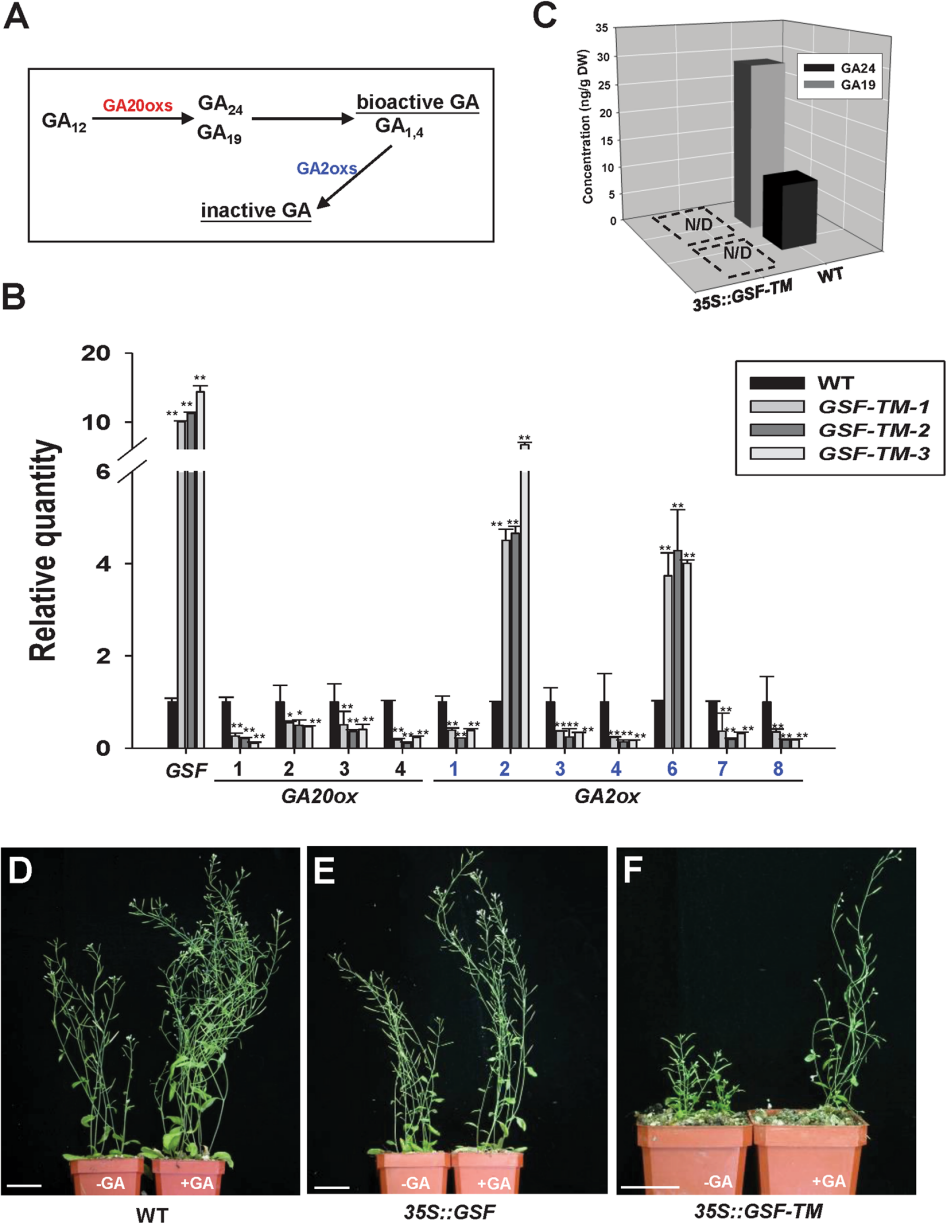
Transcript levels of GA metabolic pathway genes and the external application of GA in 35S::*GSF-TM* transgenic plants. (**A**) Schematic diagram for the gibberellin biosynthetic pathway. *GA20-oxidases* (*GA20oxs*) participate in the biosynthesis of the major bioactive GAs (GA_1_ and GA_4_) from the intermediates, such as GA_19_ and GA_24_. *GA2-oxidases* (*GA2oxs*) are involved in the late steps in the metabolic pathway to convert bioactive GAs into inactive GAs. (**B**) Detection of the expression for *GSF*, *GA20oxs (1–4)* and *GA2oxs (1,2,3,4,6,7,8)* in one wild-type (WT) plant and three severe (GSF-TM-1, 2, 3) 35S::*GSF-TM* transgenic Arabidopsis plants using real-time quantitative RT-PCR. The expression level relative to wild-type plants is presented. Error bars represent standard deviation. The asterisks indicate a significant difference from the wild type (WT) value (*P ≤ 0.05, **P ≤ 0.01) by Student’s T-test. (**C**) Detection of the concentrations of GA_19_ and GA_24_, intermediates in a bioactive GA biosynthesis pathway in wild-type (WT) and 35S::*GSF-TM Arabidopsis*. N/D indicates that a signal was not detected in the samples. (**D–F**) External supply of GA in the wild-type (**D**), 35S::*GSF* (**E**) and 35S::*GSF-TM* **(F)** plants. The shoot elongation was clearly observed in the wild-type (**D**, right) and 35S::*GSF* plants (**E**, right) after GA treatment. The significant elongation of the inflorescence (**F**, right) was also observed in the GA-treated 35S::*GSF-TM* plants. Bar = 35 mm.

It is interesting to note that the dwarfism phenotype observed in 35S::*GSF-TM* transgenic plants is similar to that of the mutants deficient in GA. It was known that gibberellin metabolic genes, such as *GA20-oxidases* (*GA20oxs*) and *GA2-oxidases* (*GA2oxs*), are involved in the late steps in the metabolic pathway^5^. The GA20oxs participated in the biosynthesis of the major bioactive GAs, while GA2oxs is important to convert GA into inactive GAs^1^ (Fig. 5A).

To determine the profile of the gibberellin metabolic genes in 35S::*GSF-TM* plants, total RNA was extracted from the whole plant of the 35S::*GSF-TM* plants, and the expression of the genes involved in GA biosynthesis was analyzed using real-time quantitative RT-PCR analysis. As expected, the *GSF* mRNA was significantly upregulated in the severe-dwarf 35S::*GSF-TM* transgenic plants (Fig. 5B). The expression level of four *GA20oxs (1–4)* examined was all significantly downregulated (Fig. 5B). Among seven *GA2oxs* examined, *GA2oxs2* and *GA2oxs6* were significantly upregulated (Fig. 5B). These results strongly suggest that the similar GA-deficient phenotype in the 35S::*GSF-TM* transgenic plants is correlated with the altered expression of the genes that participate in GA biosynthesis.

### GAs are reduced in 35S::*GSF-TM Arabidopsis* and dwarfism can be rescued by the external application of GA

Since the GA biosynthetic *GA20ox* genes were downregulated, while the GA deactivation *GA2ox2/6* genes were upregulated in 35S::*GSF-TM Arabidopsis*, it is interesting to examine whether the levels of GAs were affected in 35S::*GSF-TM Arabidopsis*. To answer this question, the concentrations of GA_19_ and GA_24_, which are two intermediates in the bioactive GA_1_ and GA_4_ biosynthetic pathway (Fig. 5A), were examined in wild-type and 35S::*GSF-TM Arabidopsis*. The result indicated that both GA_19_ and GA_24_ can only be clearly detected in wild-type plants and are undetectable in 35S::*GSF-TM Arabidopsis* (Fig. 5C). Thus, the dwarfism of 35S::*GSF-TM* transgenic *Arabidopsis* is likely to be due to the reduction of the endogenous bioactive GA.

To explore whether an external supply of GA could rescue the dwarfism of the 35S::*GSF-TM* transgenic plants, GA was applied externally to the plants. As controls, the shoot elongation was clearly observed in the wild-type (Fig. 5D) and the 35S::*GSF* plants (Fig. 5E) after GA treatment. Similarly, the elongation of the inflorescence was also observed in GA-treated 35S::*GSF-TM* plants (Fig. 5F, right), which were different from the GA-untreated short inflorescence (Fig. 5F, left). This result indicates that the dwarfism of the 35S::*GSF-TM* plants was caused by the reduction of the JA level rather than the block of the signal transduction of GA and can be rescued via the external supply of GA.

### Ectopic *GA20ox1* expression could not rescue the dwarfism in the 35S::*GSF-TM* plants

Since the expression of *GA20ox1*, which participates in the biosynthesis of the major bioactive GAs, was repressed by 35S::*GSF-TM* (Fig. 5B), 35S::*GA20ox1*/35S::*GSF-TM* double transgenic *Arabidopsis* plants were generated, and the phenotype was analyzed to further confirm their relationship. As expected, 35S::*GA20ox1* exhibited a phenotype of GA-overproduction^50^ by producing longer inflorescences than the wild-type Arabidopsis (Fig. S3A). The increasing length of the inflorescences was correlated with the expression level of *GA20ox1* (Fig. S3B).

**Figure 6:**
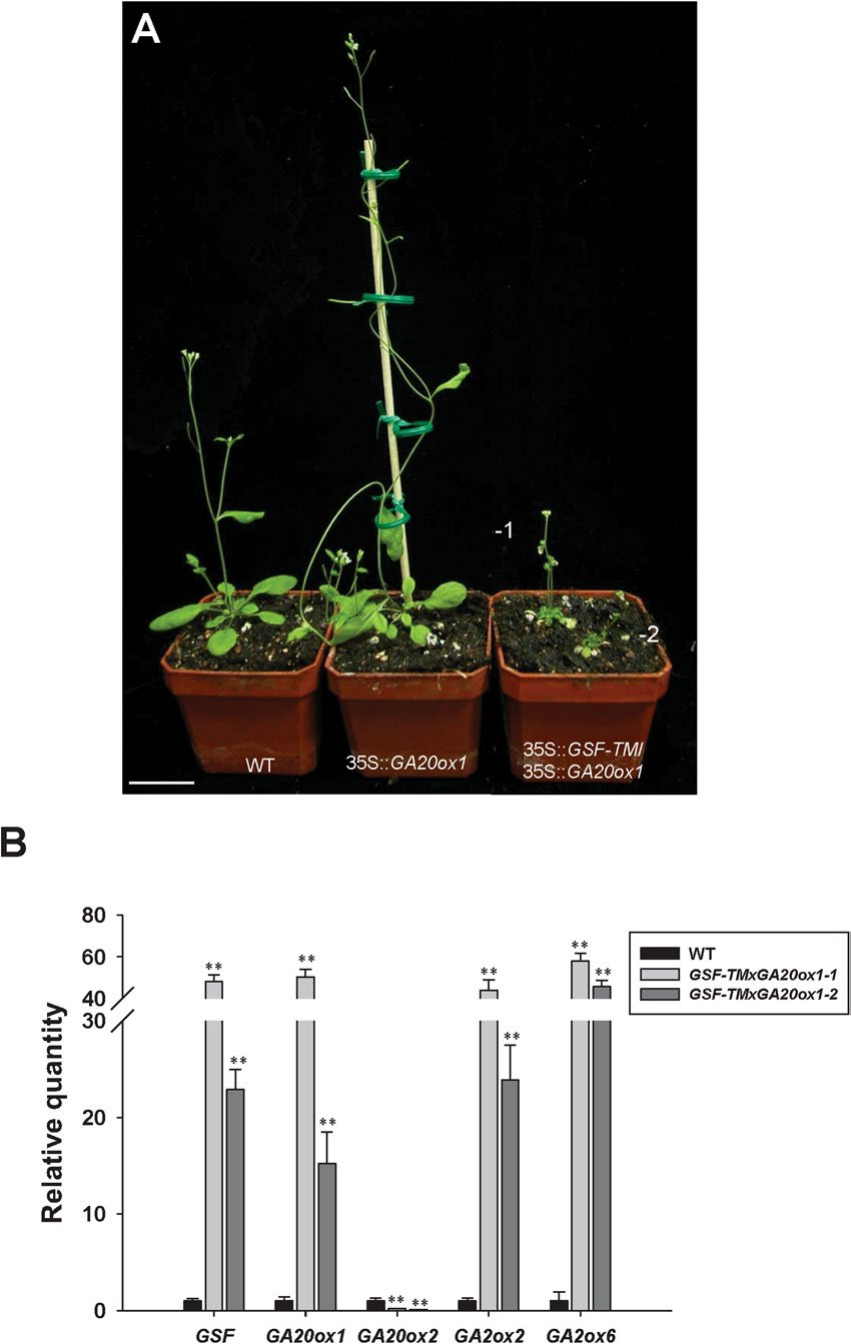
Phenotypic analysis of the 35S:*:GSF-TM*/35S::*GA20ox1 Arabidopsis* plants. (**A**) A 32-day-old 35S::*GA20ox1 Arabidopsis* plant (middle) produced longer inflorescence than a wild-type (WT) (left) Arabidopsis, while two 35S:*:GSF-TM*/35S::*GA20ox1 Arabidopsis* plants (lines 1 and 2) (right) showed a severe dwarfism phenotype with small compact leaves and a short inflorescence. Bar = 30 mm. (**B**) Detection of the expression for *GSF*, *GA20ox1, GA20ox2, GA2ox2* and *GA2ox6* in one wild-type (WT) plant and two 35S:*:GSF-TM*/35S::*GA20ox1* (lines 1 and 2) transgenic Arabidopsis plants using real-time quantitative RT-PCR. The expression level relative to wild-type plants is presented. Error bars represent standard deviation. The asterisks indicate a significant difference from the wild type (WT) value (*P ≤ 0.05, **P ≤ 0.01) by Student’s T-test.

When 35S::*GA20ox1* was introduced into 35S::*GSF-TM* to generate 35S::*GA20ox1*/35S::*GSF-TM* double transgenic Arabidopsis, the height and the size of the plants were significantly reduced to a level similar to those of the 35S::*GSF-TM* plants (Fig. 6A). As expected, the expression of *GA20ox1* in these 35S::*GA20ox1*/35S::*GSF-TM* plants was higher than that of the wild-type plants (Fig. 6B). However, the expression of *GA20ox2* was reduced, and *GA2ox2/6* was clearly upregulated by the high level of *GSF* expression (Fig. 6B). This result indicated that although the ectopic expression of *GA20ox1* exhibits a GA-overproduction phenotype, the high level of *GSF* in 35S::*GA20ox1*/35S::*GSF-TM* plants will sequentially convert the GAs into inactive ones by activating the expression of *GA2ox2/6*, resulting in a dwarfism phenotype. Thus, *GA2ox2/6* are the ultimate key factors for the ability of *GSF* to regulate the GA level in plants.

To confirm the important effect of *GA2ox2* in GA regulation, 35S::*GA2ox2* transgenic Arabidopsis plants were generated, and the phenotype analyzed. The result showed that a similar GA-deficiency dwarfism mutant phenotype, as seen in the 35S::*GSF-TM* plants, was observed in 35S::*GA2ox2* Arabidopsis (Fig. S4A–D). The severity of the dwarfism clearly correlated with the expression level of *GA2ox2* (Fig. S4E).

### Cold stress induced the translocation of GSF from the ER to the nucleus and affected the expression of *GA2oxs*

It has been reported that the expression of *GSF* was specifically upregulated in wild-type *Arabidopsis* by cold stress (4 °C)^32^. Interestingly, it has also been demonstrated that the reduction of the GA levels retarded plant growth in response to several abiotic stresses, such as cold stress^7^.

**Figure 7:**
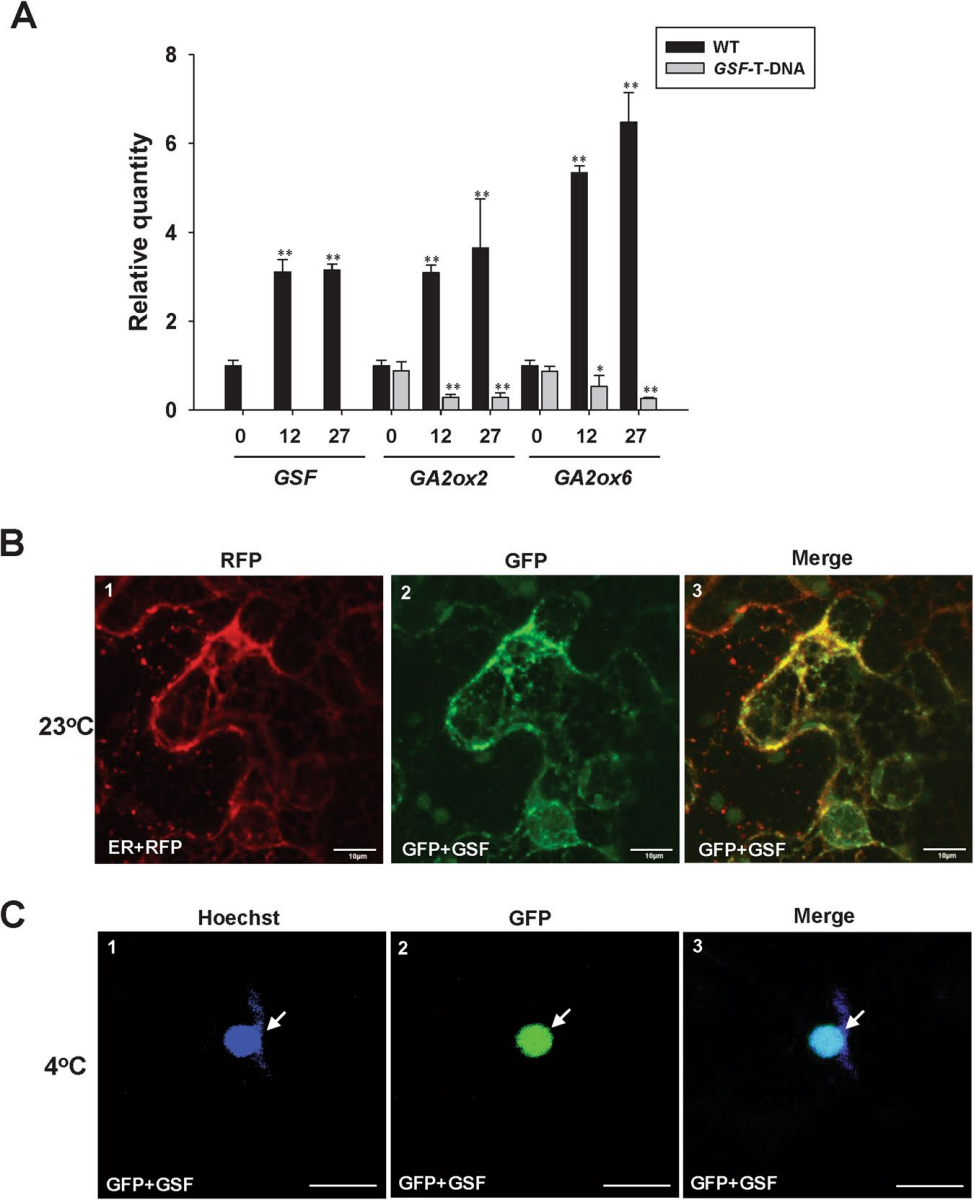
Detection of the expression for *GSF* and the GA metabolic pathway genes and the cellular localization of GSF under cold treatment. (**A**) Detection of the expression for *GSF, GA2ox2* and *GA2ox6* using real-time quantitative RT-PCR for 14-day-old wild-type *Arabidopsis* and *GSF* T-DNA mutants (SALK_022174) after exposure to 4 °C over a period of 12 and 27 hours, respectively. For the detection of *GSF* expression in SALK_022174 mutant, the primers pair F-1 (GSF qRT for-1) and R-1 (GSF qRT rev-1) (Supplementary Table S1), which located in the two sides of the T-DNA insertion (Fig. S6), were used. The expression level relative to wild-type plants is presented. Error bars represent standard deviation. The asterisks indicate a significant difference from the untreated wild type (WT, time 0) value (*P ≤ 0.05, **P ≤ 0.01) by Student’s T-test. (**B**) Agrobacterium-mediated transient expression of GFP + GSF and ER + RFP in the epidermal cells of *N. benthamiana* at 23 °C. A GFP + GSF fusion protein (−2) accumulated in the ER where the RFP was localized (−1). A merged fluorescence image of (−1, −2) in (−3) showing the similar localization of GFP + GSF and ER + RFP. Scale bars: 10 μm. (**C**) Agrobacterium-mediated transient expression of GFP + GSF in the epidermal cells of *N. benthamiana* at 23 °C for 2 days and exposed at 4 °C (cold stress) for 6 hours. GFP + GSF fusion protein (−2) accumulated in the nucleus (arrow) where blue DNA-staining by Hoechst (arrow) was localized (−1). A merged fluorescence image of (−1, −2) in (−3) showing the similar localization (arrow) of GFP + GSF and blue DNA-staining (Hoechst). Scale bars: 10 μm.

To explore the relationship between cold stress and GSF-GA, 14-day-old wild-type *Arabidopsis* seedlings were exposed to 4 °C over a period of 12 and 27 h, respectively, and the gene expression was analyzed. The result indicated that the transcript level of *GSF* was gradually upregulated after 12-27-h treatment (Fig. 7A). When *GA2ox2* and *6*, were examined, their expression was constantly induced after cold treatment (Fig. 7A). In contrast, the expression of *GA20oxs (1–4)* was significantly down-regulated after 27-h treatment (Fig. S5). When the *GA2ox2* and 6 were examined in the *GSF* T-DNA insertion mutants (SALK_022174), which showed completely abolished expression of *GSF* (Fig. 7A), both *GA2ox2* and 6 expression was significantly down-regulated compared to that in wild-type plants (Fig. 7A) and was constantly reduced after cold treatment (Fig. 7A). These results revealed that GSF is likely to be involved in the response to cold stress by regulating the GA level primarily through the activation of *GA2ox2/6* expression. Without *GSF* function, *GA2ox2/6* expression was not induced in *GSF* T-DNA insertion mutants.

Our data showed that GSF requires translocation from the ER to the nucleus to perform its functions (Figs. 3 and 4). To identify whether cold stress influences this GSF translocation, GFP + GSF and ER-RFP were coexpressed in tobacco leaves, and their fluorescence images were analyzed after cold (4 °C) treatment. The fluorescent images of GFP + GSF fusion proteins were localized in ER and absent in the nuclei at 23 °C (Fig. 7B). In contrast, the visualization of GFP fluorescence merged with the blue fluorescence of Hoechst (a dye for DNA staining) in some cells revealed that the GFP + GSF fusion proteins were clearly located in the nucleus during 4 °C treatment (Fig. 7C). Thus, we confirmed that the plants respond to cold stress by inducing the translocation of GSF from the ER into the nucleus, which will cause the suppression of the GA level and retard plant growth.

### 35S::*GSF-TM* transgenic plants exhibit tolerance to drought stress

**Figure 8:**
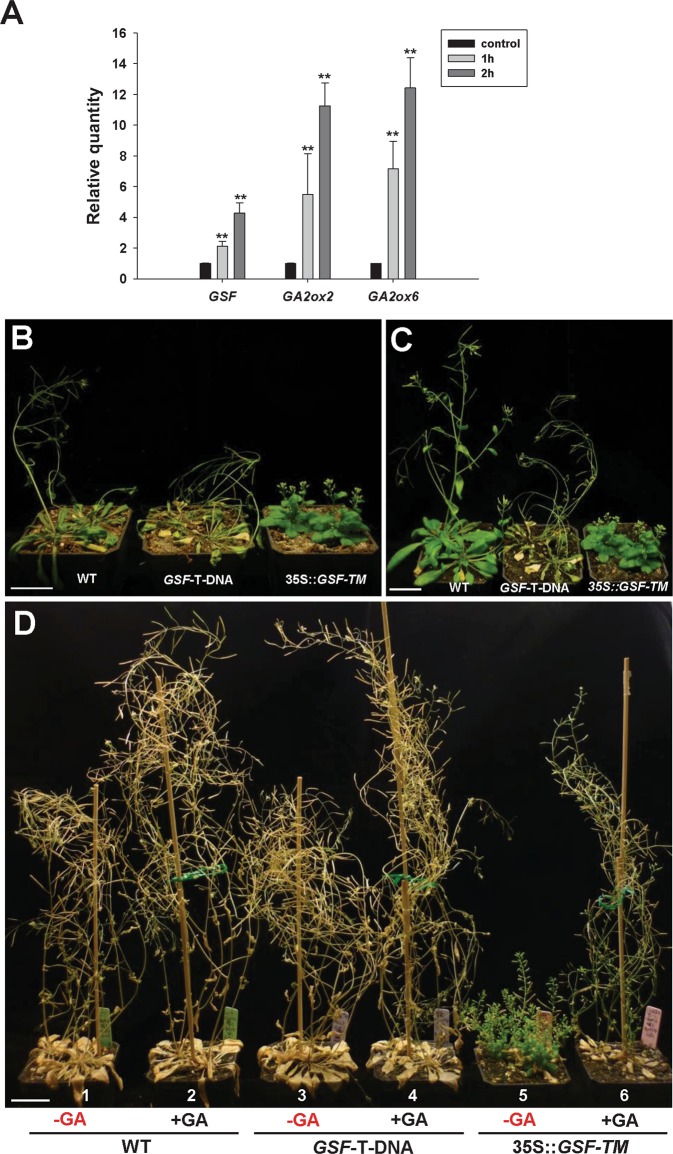
Phenotypic analysis of the 35S::*GSF-TM* and *GSF* T-DNA mutant *Arabidopsis* plants under drought treatment. (**A**) Detection of the expression for *GSF, GA2ox2* and *GA2ox6* using real-time quantitative RT-PCR on 21-day-old wild-type *Arabidopsis* after pulling the plant from the soil and exposing directly to air for 1 and 2 hours, respectively. The expression level relative to untreated control plants is presented. Error bars represent standard deviation. The asterisks indicate a significant difference from the wild type (WT) value (*P ≤ 0.05, **P ≤ 0.01) by Student’s T-test. (**B**) 28-day-old wild-type plant (left) showed a wilt phenotype and a more severe wilt phenotype was observed in *GSF* T-DNA insertion mutant (middle), while the 35S::*GSF-TM* plants (right) still grew normally without showing signs of wilt after growth under drought conditions (without irrigation) for 12 days. Bar = 30 mm. (**C**) When the plants in (**B**) were further irrigated for 5 days, a clearly recovery from wilt phenotype was observed for wild-type plant (left) whereas a severe wilt phenotype was still observed in *GSF* T-DNA insertion mutant (middle). Bar = 30 mm. (**D**) 40-day-old GA-treated wild-type plants (−2) and *GSF* T-DNA insertion mutant (−4), which showed shoot elongation, and GA-nontreated wild-type plants (−1) and *GSF* T-DNA insertion mutant (−3) showed a similar wilt phenotype after growth at drought condition for 12 days. 40-day-old GA-treated 35S::*GSF-TM* plants with shoot elongation showed a wilt phenotype (−6), while the GA-untreated 35S::*GSF-TM* plants (−5) still grew normally without showing signs of wilt after growth under drought conditions for 12 days. Bar = 30 mm.

It has been reported that the expression of *GSF* was also up-regulated in wild-type *Arabidopsis* by drought stress^32^. To explore the relationship between drought stress and GSF/GA, four-week-old wild-type *Arabidopsis* seedlings were exposed to drought conditions by removing the plants from the soil and exposing them directly to the air for a period of 1 and 2 h, respectively, and the gene expression was analyzed. The results indicated that the transcript level of *GSF, GA2ox2* and 6 was clearly up-regulated after 1- and 2-h treatments (Fig. 8A). These results revealed that GSF is also likely to be involved in the response to drought stress by regulating the GA level primarily through the activation of *GA2ox2/6* expression.

We further test the relationship among *GSF*, GA and drought tolerance in wild-type, 35S::*GSF-TM* and *GSF* T-DNA mutant plants. When four weeks old wild-type *Arabidopsis* were grown at drought condition (without irrigation) for 12 days, a clearly wilt phenotype was observed (Fig. 8B, left). Interestingly, a more severe wilt phenotype was observed in *GSF* T-DNA insertion mutant (Fig. 8B, middle) which was indistinguishable from wild-type plants when grown in normal condition. By contrast, 35S::*GSF-TM* plants still grew normally without showing sign of wilt (Fig. 8B, right). When the plants in Fig. 8B were further irrigated for 5 days, a clearly recovery from wilt phenotype was observed for wild-type plant (Fig. 8C, left). In contrast, a severe wilt phenotype was still observed in *GSF* T-DNA insertion mutant (Fig. 8C, middle). These results revealed that GSF is likely to be involved in the tolerance to drought stress by activating the *GA2ox2/6* expression. The tolerance to drought was clearly reduced in *GSF* T-DNA insertion mutants due to the abolishment of the *GSF* function.

When GA-treated wild-type plants which showed shoot elongation were grown at drought condition for 12 days, similar wilt phenotype was observed in these GA-treated (Fig. 8D–2) as in un-treated wild-type plants (Fig. 8D–1). Similar result was observed in GA-treated (Fig. 8D–4) as in un-treated *GSF* T-DNA insertion mutants (Fig. 8D–3). Interestingly, the tolerance to drought as seen in GA-untreated 35S::*GSF-TM* plants (Fig. 8D–5) was significantly altered in GA-treated 35S::*GSF-TM* plants (Fig. 8D–6) which showed wilt phenotype after 12 days grown at drought condition. This result supported that the low level of GA in 35S::*GSF-TM* plants could enhance the drought tolerance which was reduced after externally applying GA.

## Discussion

Plant-specific NAC proteins are commonly identified in various plant species, suggesting that they participate in the regulation of many aspects of plant development. In this study, the *NAC*-like gene *GSF* was characterized and functionally analyzed in *Arabidopsis*. GSF acts as a transcriptional activator based on the activation assay in yeast. The transacting domain was found to be allocated in the C-terminal half of the GSF proteins, since GSF^1–160^ (truncated C terminal half) completely abolished the activation function, while the GSF^161–521^ (truncated N terminal half) remained as active as the full-length GSF. The nature of GSF as a transcriptional activator was further supported by the result that a similar altered dwarfism phenotype was observed in 35S*::GSF-TM* and 35S*::GSF-TM* + *VP16* transgenic Arabidopsis.

It has been reported that a group of membrane-bound NAC transcription factors (NTLs) are transported into the nucleus to regulate the expression of downstream genes after their release from the ER^31^. Not surprisingly, we found that only GFP + GSF-TM fusion proteins accumulate in the nuclei of the cells, while the GFP + GSF fusion proteins accumulated in the ER and were absent in the nuclei. Thus, it is reasonable to propose that GSF requires processing from the ER and entry into the nucleus to perform its function. This assumption was further supported by the fact that only 35S*::GSF-TM* caused an altered phenotype in transgenic Arabidopsis, while 35S*::GSF* Arabidopsis was phenotypically indistinguishable from the wild-type plants.

To further investigate the function of *GSF*, the altered phenotype of 35S*::GSF-TM* Arabidopsis was analyzed. The ectopic expression of *GSF-TM* caused a dwarfism phenotype with compact and curled rosettes and short inflorescence internodes that were similar to those in the plants with GA-deficiency. Thus, this finding revealed that the function of the *GSF* gene is likely to be related to the modulation of GA biosynthesis or signal transduction in plants. Several lines of evidence provided additional evidence for the hypothesis that GA biosynthesis rather than the signal transduction of GA was regulated by *GSF*. First, when the expression of *GA20oxs* and *GA2oxs*, which are involved in late steps in the metabolic pathway^5^ were tested, a clear downregulation of *GA20oxs (1–4)* and upregulation of *GA2oxs2* and *GA2oxs6* were observed in 35S*::GSF-TM* Arabidopsis. Since the GA20oxs had participated in the biosynthesis of the major bioactive GAs, while GA2oxs functions to convert GA into inactive GAs^1^, the repression of *GA20oxs (1–4)* and activation of *GA2oxs (2, 6)* are expected to decrease the level of the bioactive GAs in 35S*::GSF-TM* Arabidopsis. Interestingly, we found that 35S::*GA20ox1* could not rescue the dwarfism of the 35S::*GSF-TM* plants. This indicated that the products of GA-overproduction by 35S::*GA20ox1* will be sequentially converted into inactive GAs by the high activity of *GA2ox2/6* in the 35S::*GA20ox1*/35S::*GSF-TM* double transgenic plants. The second line of evidence results from the almost undetectable concentrations of GA_19_ and GA_24_, two intermediates in the bioactive GA biosynthesis pathway, in 35S*::GSF-TM* Arabidopsis. Third, an external supply of GA rescued the dwarfism of the 35S::*GSF-TM* transgenic plants. Thus, the dwarfism observed in 35S::*GSF-TM* transgenic *Arabidopsis* is likely to be caused by the reduction of endogenous bioactive GA due to the high level of functional GSF-TM in the nucleus.

The next question is what mechanisms *GSF* participated in during the regulation of GA activity. It is worth noting that the reduction of the GA levels retarded plant growth in response to several abiotic stresses^7^. For example, the overexpression of *DDF1* causes dwarfism in *Arabidopsis* by upregulating the expression of *GA2ox7* and reducing the contents of bioactive GA under high-salinity stress^11^. It has been reported that the level of several *GA2oxs* was upregulated, while *GA20oxs* was downregulated in response to cold stress^9,12^. However, the mechanisms of the regulation of the GA level during cold or drought stress remained to be investigated. Interestingly, *GSF* has also been reported^32^ and was demonstrated in this study to be upregulated in wild-type *Arabidopsis* by cold and drought stresses. Thus, *GSF* is likely to be a link between *GSF* and GA-related cold/drought response. This assumption was clearly supported by the results that GSF can be processed from the ER and enter the nucleus under cold treatment, as well as the exhibition of the tolerance to drought stress by the 35S::*GSF-TM* transgenic plants. All these results indicated that *GSF* functions to reduce the GA level in response to the cold/drought stresses in plants.

**Figure 9:**
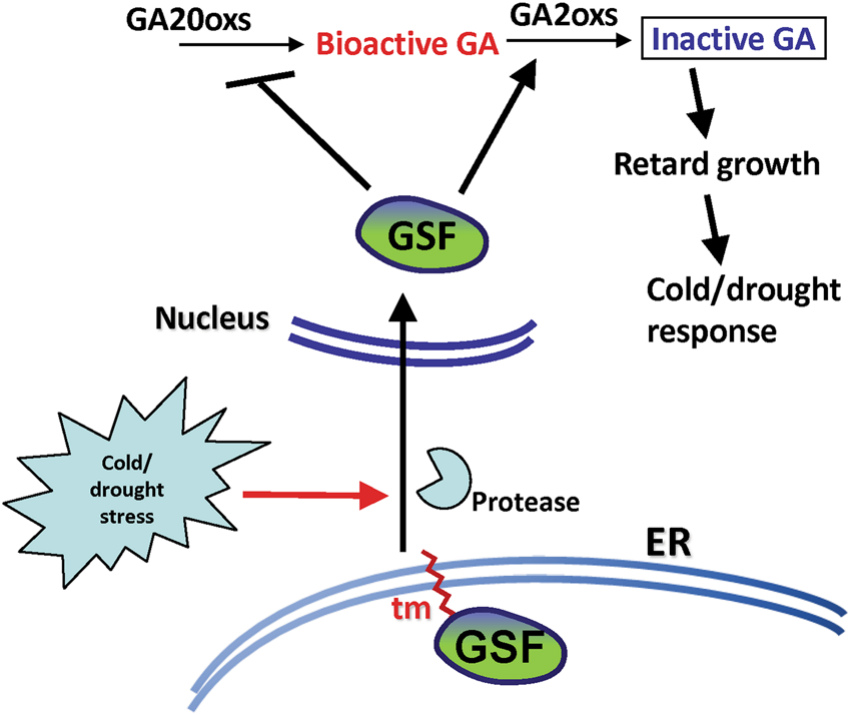
Model for the function of *GSF* in regulating gibberellin biosynthesis in response to cold/drought stresses in *Arabidopsis*. In the wild-type plants, the GSP proteins are localized at the membrane of ER through the transmembrane motif (tm) when grown under normal conditions. The cold/drought stresses activate the *GSF* expression and enhanced the processing of GSF by protease from the ER membrane, and it will enter the nucleus. The functional form of GSF-TM will repress *GA20oxs* and activate *GA2oxs* expression to convert the bioactive form of GA to the inactive form, resulting in the retardation of the plant growth in response to stress. 35S::*GSF-TM* suppressed the bioactive form of the GA level during the whole plant development and resulted in a dwarfism phenotype.

As illustrated in Fig. 9, our results reveal a possible model for the interaction of the *GSF* and GA in the regulation of plant growth and development. In *Arabidopsis*, the GSF proteins targeted to the ER membrane through the transmembrane motif (Fig. 3) when grown under normal conditions. Abiotic stresses, such as cold/drought, not only activated the *GSF* expression but also significantly enhanced the processing of GSF by a protease from the ER membrane and its entry into the nucleus to perform its function. This effect repressed *GA20oxs* and activated *GA2oxs* expression, eventually suppressing the biosynthesis of bioactive form of GA and retarding the plant growth in response to stress. The ectopic expression of *GSF-TM* continuously enhanced the reduction of the GA level during all of the stages of plant growth and resulted in a dwarfism phenotype as seen in our results. Thus, our discovery uncovered the involvement of the *GSF* gene in a mechanism that regulated the GA level during cold or drought stress.

## Materials and Methods

### Plant materials and growth conditions

The T-DNA insertion mutants of *GSF* (SALK_022174) *Arabidopsis* seeds were obtained from the Arabidopsis Biological Resource Center, Ohio State University, Columbus, OH, USA. The seeds for *Arabidopsis* were sterilized and placed on agar plates containing 1/2 X MS media^51^ at 4 ^°^C for 2 days. The seedlings were grown in growth chambers under long-day conditions (16-h light/8-h dark) at 22 °C for 10 days before being transplanted to the soil. The light intensity of the growth chambers was 150 μE m^−2^ s^−1^.

### Cloning of *Arabidopsis GSF* cDNA

The *Arabidopsis GSF* (At1g34190), containing four exons and three introns, was identified on chromosome one. A cDNA containing an open reading frame of *GSF* was amplified by RT-PCR using the 5′ primer, GSF-for and the 3′ primer, GSF-rev. cDNA truncated with the transmembrane motif region of *GSF*-*TM* was amplified by RT-PCR using the 5′ primer, GSF-for, and the 3′ primer, GSF-TM-rev. All of the primers contained the generated *Kpn*I recognition site (5′-GGTACC-3′) and the *Sac*I recognition site (5′-GAGCTC-3′) to facilitate the cloning of the *GSF* cDNA. Sequences for the primers are listed in Supplementary Table S1. The amplified *Kpn*I-*Sac*I fragments containing either the full-length or truncated transmembrane motif region for the *GSF* gene was cloned into the linker region in the binary vector pBImGFP2 (CHY Lab, Taichung, Taiwan, unpublished) under the control of the cauliflower mosaic virus (CaMV) 35S-Ω promoter (35S::*GSF* and 35S::*GSF-TM)* and used for plant transformations.

### Construction of the *GSF-TM* + *VP16* construct

To generate the *GSF-TM* + *VP16* construct, the cDNA for *GSF-TM* was obtained by PCR amplification using the GSF-for-KpnI and GSF-TM primers that contained the *Kpn*I recognition sites to facilitate the cloning of the cDNA. The PCR fragment containing the *GSF-TM* was cloned into the pEpyon-3bK plasmid^29^ in front of the VP16-AD sequence and under the control of the CaMV 35S promoter and was used for plant transformation. The sequences for the primers are listed in the Supplementary Table S1.

### GSF*::GUS* fusion construct

To generate the *GSF*::*GUS* construct, the *GSF* promoter (2.2 kb) was obtained by PCR amplification from the genomic DNA using the GSF-Pro-for and GSF-Pro-rev primers and cloned into the pGEMT easy vector (Promega, Madison, WI, USA). This 2.2 kb fragment for the *GSF* promoter was subcloned into the linker region before the β-Glucuronidase (GUS) coding region in the binary vector pBI101 (CLONTECH, Palo Alto, CA, USA). The primers contained the generated *Pst*I (5′-CTCCAG-3′) and *Xba*I (5′-GGATCC-3′) recognition sites to facilitate the cloning of the promoter. The sequences for the primers are listed in Supplementary Table S1.

### Construction of the 35S::*GA2ox2* construct

To generate the 35S::*GA2ox2* construct, the cDNA for *GA2ox2* was obtained by PCR amplification using the AtGA2ox2 for PstI and AtGA2ox2 rev SalI primers that contained the generated *Pst*I recognition site (5′-CTGCAG-3′) and *Sal*I recognition site (5′-GTCGAC-3′) to facilitate the cloning of the cDNA. The PCR fragment containing the *GA2ox2* was cloned into the linker region in the binary vector pEpyon-32K (CHY Lab, Taichung, Taiwan, unpublished) under the control of cauliflower mosaic virus (CaMV) 35S-Ω promoter (35S::*GA2ox2)* and used for plant transformation. The sequences for the primers are listed in the Supplementary Table S1.

### Construction of the 35S::*GA20ox1* construct

To generate the 35S::*GA20ox1* construct, the cDNA for *GA20ox1* was obtained by PCR amplification using the 5′ primer, At4g25420 (GA20ox1)-for and the 3′ primer, At4g25420 (GA20ox1)-rev primers that contained the generated *Kpn*I recognition site (5′-GGTACC-3′) and *Sac*I recognition site (5′-GAGCTC-3′) to facilitate the cloning of the cDNA. The PCR fragment containing the *GA20ox1* was cloned into the linker region in the binary vector pEpyon-32K (CHY Lab, Taichung, Taiwan, unpublished) under the control of the cauliflower mosaic virus (CaMV) 35S-Ω promoter (35S::*GA20ox1)* and used for plant transformation. To generate 35S::*GA20ox1/*35S::*GSF-TM Arabidopsis*, 35S::*GA20ox1 Arabidopsis* (homozygote) was transformed with 35S::*GSF-TM* to generate 35S::*GA20ox1/*35S::*GSF-TM Arabidopsis*. The sequences for the primers are listed in the Supplementary Table S1.

### GSF transcriptional activation assay in yeast

The full length and various truncated cDNA fragments for *GSF* were amplified using RT-PCR: GAL4BD-GSF^1~557^ (primer pair: GSF-for-atg-Y + GSF N-557 rev), GAL4BD-GSF^1~521^ (primer pair: GSF-for-atg-Y + GSF N-521 rev), GAL4BD-GSF^161~521^ (primer pair: GSF N-161 for + GSF N-521 rev), GAL4BD-GSF^1~160^ (primer pair: GSF-for-atg-Y + GSF N-160 rev) and a positive control GAL4AD (primer pair: GAL4 AD for + GAL4 AD rev). The sequences for the primers are listed in Supplementary Table S1. These amplified fragments were integrated into the *Nde*I–*Sal*I site of pGBKT7 (CLONTECH, Palo Alto, CA, USA), containing the GAL4 DNA-binding domain, to produce the fusion proteins GAL4BD-GSF^1~557^, GAL4BD-GSF^1~521^, GAL4BD-GSF^161~521^ and GAL4BD-GSF^1~160^. The pGBKT7 harboring different fusion proteins was introduced into the yeast strain AH109, which contained four reporter genes (*lacZ*, *His3*, *ADE2* and *MEL1*). The transformed yeast cells were selected using synthetic dropout (SD) plates lacking tryptophan at 30 °C for 3 days. Simultaneously, the transformed yeast cells were grown on SD plates lacking tryptophan, histidine and adenine at 30 °C for 3 days and used to quantify the β-galactosidase activity as described previously^52^.

### Real-time PCR analysis

For real-time quantitative RT-PCR, total RNA was isolated from wild-type (WT) and transgenic plants and used as templates. The transcript levels for each genes were determined using three biological replicates and normalized against *UBQ10*. The RT-PCR reaction was performed on an MJ Opticon system (MJ Research, Waltham, MA) using a SYBER Green Real-time PCR Master Mix (TOYOBO Co., LTD.). The amplification condition was 95 °C for 10 minutes, followed by 40 cycles of amplification (95 °C for 15 seconds, 58 °C for 15 seconds, 72 °C for 30 seconds followed by plate reading) and melting (50–95 °C with plate readings every 1 °C). Sequences for the primers used for real-time quantitative RT-PCR for *GSF, GA20ox1*, *GA20ox2*, *GA20ox3, GA20ox4, GA2ox1, GA2ox2, GA2ox3, GA2ox4, GA2ox6, GA2ox7* and *GA2ox8* are listed in Supplementary Table S1. The housekeeping gene *UBQ10* was used as a normalization control with the following primers: RT-UBQ10-F and RT-UBQ10-4-2. All the experiments were repeated at least twice. The data were analyzed using the Gene Expression Macro software (Version 1.1, Bio-Rad).

### Plant transformation and transgenic plant analysis

Constructs generated in this study were introduced into *Agrobacterium tumefaciens* strain *GV3101* and transformed into *Arabidopsis* plants using the floral dip method as previously described^53^. Transformants that survived in the media containing kanamycin (50 µg mL^−1^) were verified using RT-PCR analysis.

### Histochemical GUS assay

Histochemical staining was performed using the standard method described previously^54,55^.

### Scanning electron microscopy (SEM)

Scanning electron microscopy was performed as described previously^52,56^. Samples were fixed in 2% glutaraldehyde in 25 mM sodium phosphate buffer (pH 6.8) at 4 ^°^C overnight. After dehydration in a graded ethanol series, the specimens were dried to their critical point in liquid CO_2_. The dried materials were mounted and coated with gold-palladium in a JBS sputter-coater (model 5150). Specimens were examined using a Field emission scanning electron microscope (JEOL JSM-6700F, Japan) with an accelerating voltage of 15 kV.

### Construction of the *GFP* + *GSF* and *GFP* + *GSF-TM* constructs

For the localization assay of the GSF protein, *GFP* + *GSF* and *GFP* + *GSF-TM* constructs were constructed. The *GFP* cDNA without a stop codon was amplified from a pBI-mGFP2 expression vector, while connecting 30 base pairs that encode a 5X Gly-Ala linker at the 3′ end of *GFP* using two step PCR^57^. The short oligonucleotide fragment of GFP-Gly-Ala was amplified using the 5′ primer FmGFP5L-for (*Kpn*I) and the inner 3′ primer FmGFP5L-rve I. The full oligonucleotide fragment of GFP-Gly-Ala was amplified using the 5′ primer FmGFP5L-for (*Kpn*I) and the inner 3′ primer FmGFP5L-rev II (*Hind*III). The *GSF* or *GSF-TM* cDNA with the stop codon was in frame to the 3′ end of GFP-Gly-Ala, respectively, using GSF-for-atg (*Hind*III) and fuse-GSF-rev for *GSF*, 5′ primer GSF-for-atg (*Hind*III) and the 3′ primer GSF-TM-rev (*Sac*I) for *GSF-TM*. The sequences for the primers are listed in Supplementary Table S1. ER-RFP, which contained the ER marker fused with the RFP fluorescent protein, was obtained from the ABRC (clone name: ER-RK).

### Transient expression assay of *Nicotiana benthamiana*

Fully expanded young leaves from 4-week-old *Nicotiana benthamiana* plants were infiltrated with the *Agrobacterium tumefaciens* strain C58C1 that contained the 35S::*GSF* + *GFP* or 35S::*GSF-TM* + *GFP* fusions as previously described^58^. The signal in the infiltrated leaves was observed using an Olympus FV1000 confocal microscope (Olympus FV1000, Tokyo, Japan). Hoechst 33342 (0.02 mM) that was excited at 405 nm with the laser; the GFP was excited at 488 nm, and the RFP was excited at 543 nm.

### Quantification of GA intermediates level

To measure the GA intermediates at the GA_19_ and GA_24_ level, 0.5 g frozen plant sample of Arabidopsis was sent to the Plant Biotechnology Institute (PBI), National Research Council (NRC), Canada for the additional extraction, purification and quantification of the GA intermediates (https://www.nrc-cnrc.gc.ca/eng/solutions/advisory/plant_hormone.html).

### Application of GA_3_

To determine whether the plants were responsive to gibberellin, a modified method described previously^59^ was applied by spraying 100 *µ*M GA_3_ (Sigma) dissolved in 100% absolute ethanol to the plant grown on soil.

## Supplementary information

Supplementary Information
