# Supplementary material for: NAC-Like Gene GIBBERELLIN SUPPRESSING FACTOR Regulates the Gibberellin Metabolic Pathway in Response to Cold and Drought Stresses in Arabidopsis
Source: Sci Rep. 2019 Dec 17;9:19226. doi: 10.1038/s41598-019-55429-8 (PMC6917810; doi:10.1038/s41598-019-55429-8)
Supplement: Supplementary file 1 — Supplementary Information [file 41598_2019_55429_MOESM1_ESM.pdf]

NAC-Like Gene *GIBBERELLIN SUPPRESSING FACTOR* Regulates the Gibberellin Metabolic Pathway in Response to Cold and Drought Stresses in *Arabidopsis*

Hong-Ie Chen[1], Pei-Fang Li[1] and Chang-Hsien Yang[1,2]*

[1]Institute of Biotechnology,
[2]Advanced Plant Biotechnology Center,
National Chung Hsing University,
Taichung, Taiwan 40227 ROC

* To whom correspondence should be addressed
E-mail: chyang@dragon.nchu.edu.tw

Chen_Fig.S1

A

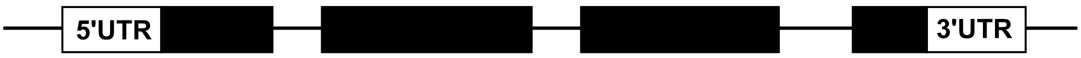

B

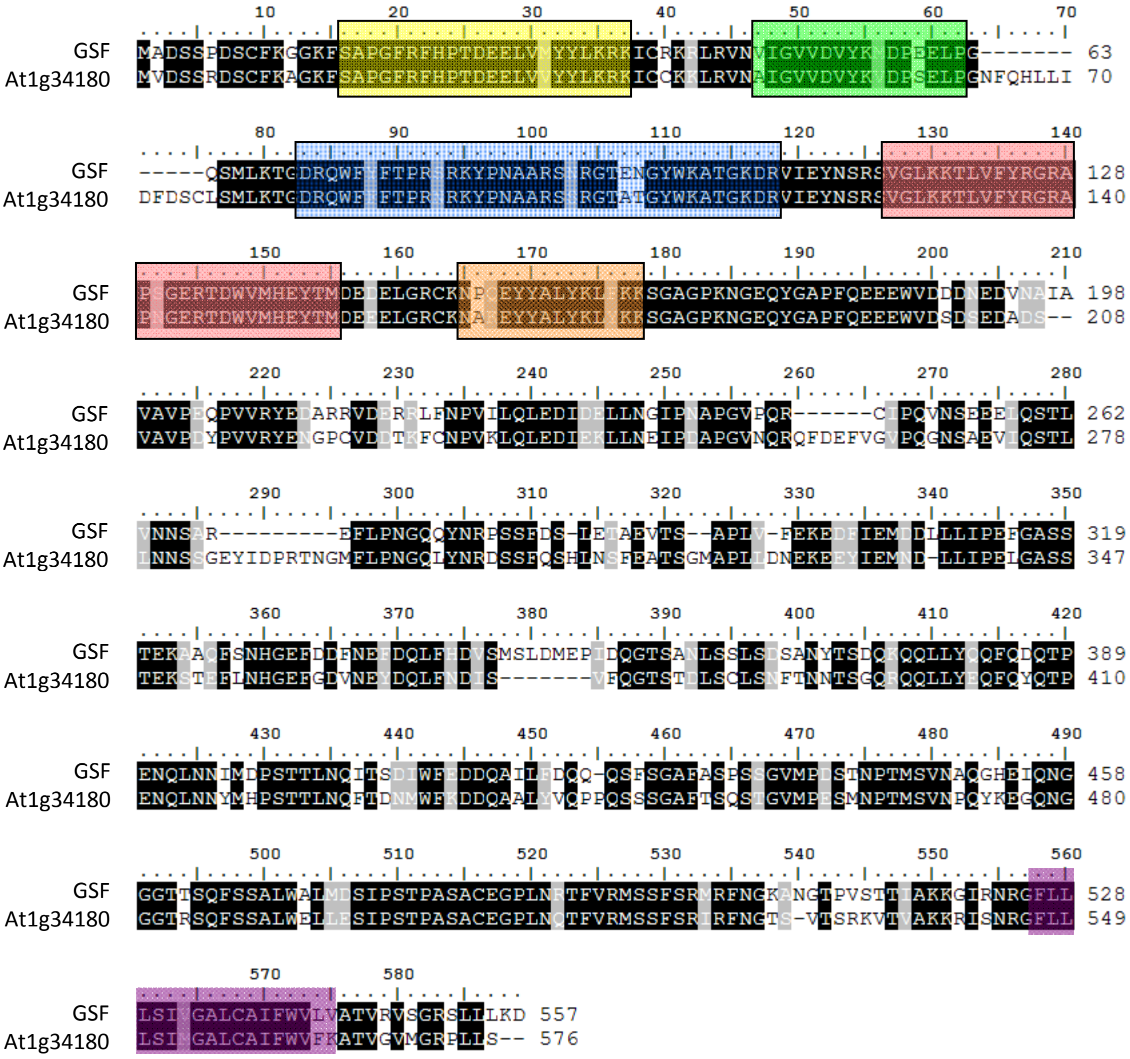

Supplementary Figure S1. Gene structure and protein sequence of *Arabidopsis GSF*.
(A) Schematic diagram for the *GSF* gene, which contains 3 introns (line) and 4 exons (black boxes). 5'UTR and 3'UTP indicates the 3' and 5' untranslated regions, respectively.
(B) Protein sequence comparison of GSF (At1g34190) and the most closely related *Arabidopsis* NAC-like protein At1g34180. The GSF protein contains a putative conserved NAC domain in the N terminal that consists of five subdomains (A-E; A: yellow box, B: green box, C: blue box, D: red box, E: orange box). In the C terminus, one transmembrane motif is indicated in the purple box. In each alignment, identical or similar amino acid residues are indicated by black or gray boxes, respectively. Dashes were introduced to improve alignment. This sequence alignment was generated using the Clustal W-Multiple Sequence Alignment Program at the Biology Work Bench (http://workbench.sdsc.edu).

Chen_Fig.S2

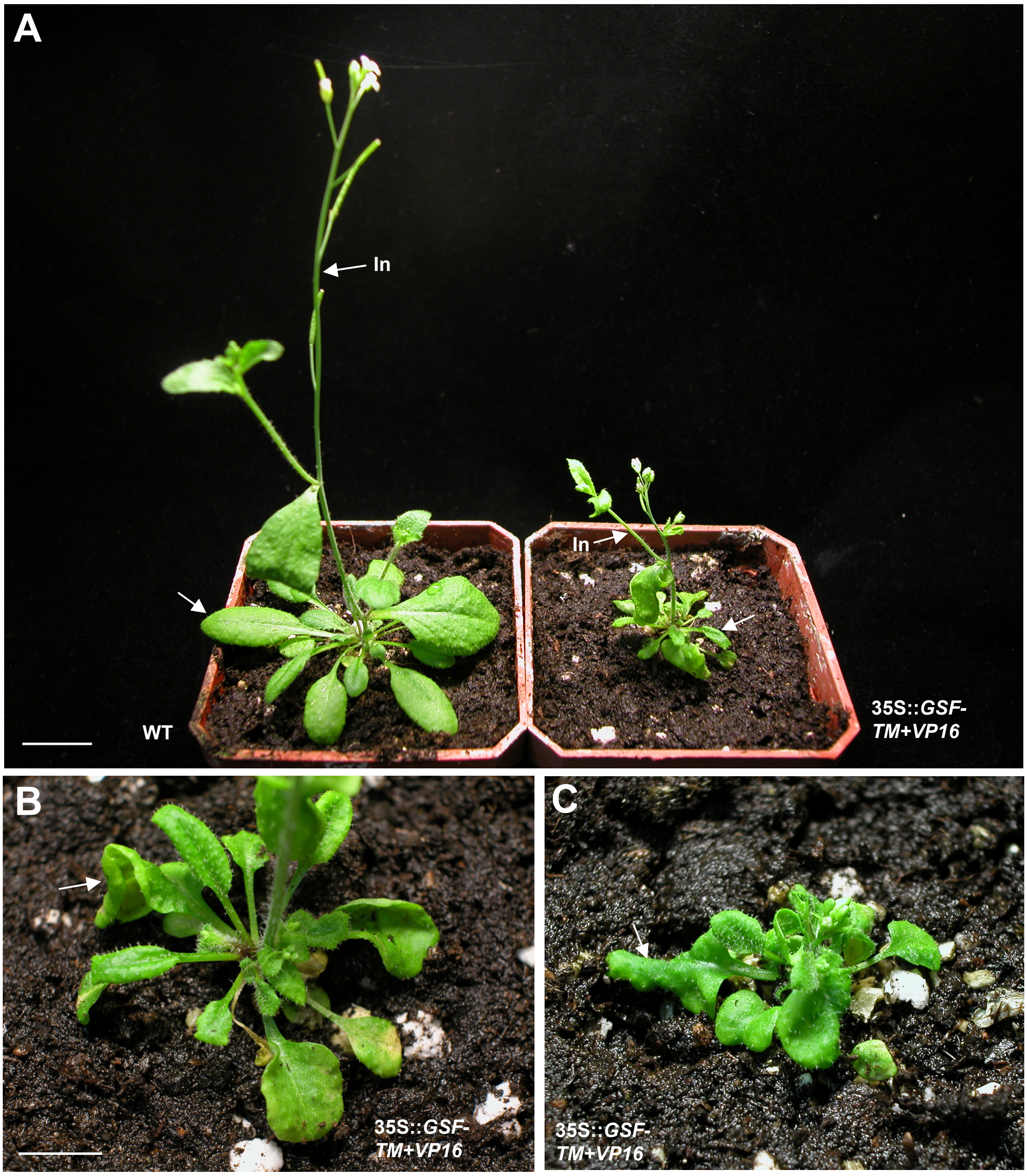

Supplementary Figure S2. Phenotypic analysis of the 35S::*GSF-TM+VP16 Arabidopsis* plants.
(A) A 32-day-old 35S::*GSF-TM+VP16* plant (right) showed a severe dwarfism phenotype with small compact leaves (arrow) and short inflorescence (In), while wild-type plants (WT, left) produced normal rosette leaves (arrow) and well-elongated inflorescence (In). Bar = 15mm
(B-C) Close-up of two 35S::*GSF-TM+VP16* plants with small compact and curled leaves (arrow). Bar = 5 mm.

Chen_Fig.S3

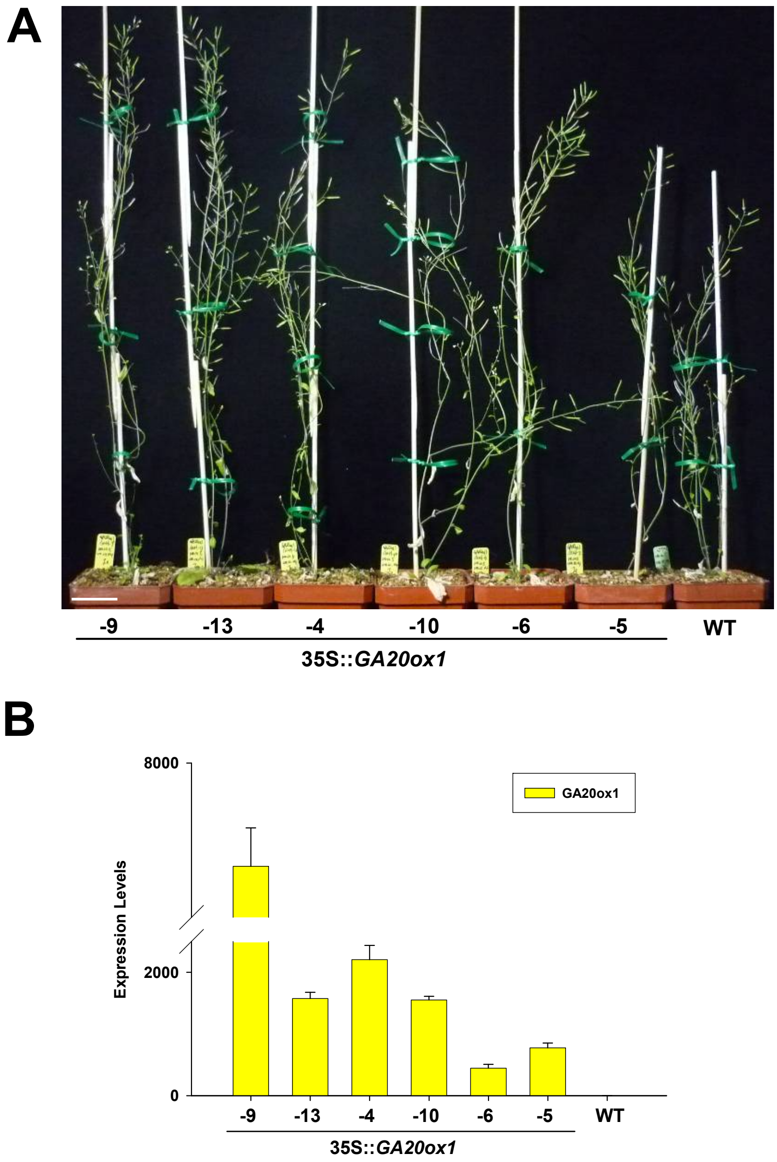

Supplementary Figure S3. Phenotypic analysis of the 35S::*GA20ox1 Arabidopsis* plants.
(A) Six 35S::*GA20ox1 Arabidopsis* plants (lines 9, 13, 4, 10, 6 and 5) produced a longer inflorescence than the wild-type (WT) Arabidopsis similar to the GA-overproduction phenotype. Bar = 30 mm.
(B) The detection of the expression level of *GA20ox1* in six 35S::*GA20ox1 Arabidopsis* plants and one wild-type (WT) plant using real-time quantitative RT-PCR.

Chen_Fig.S4

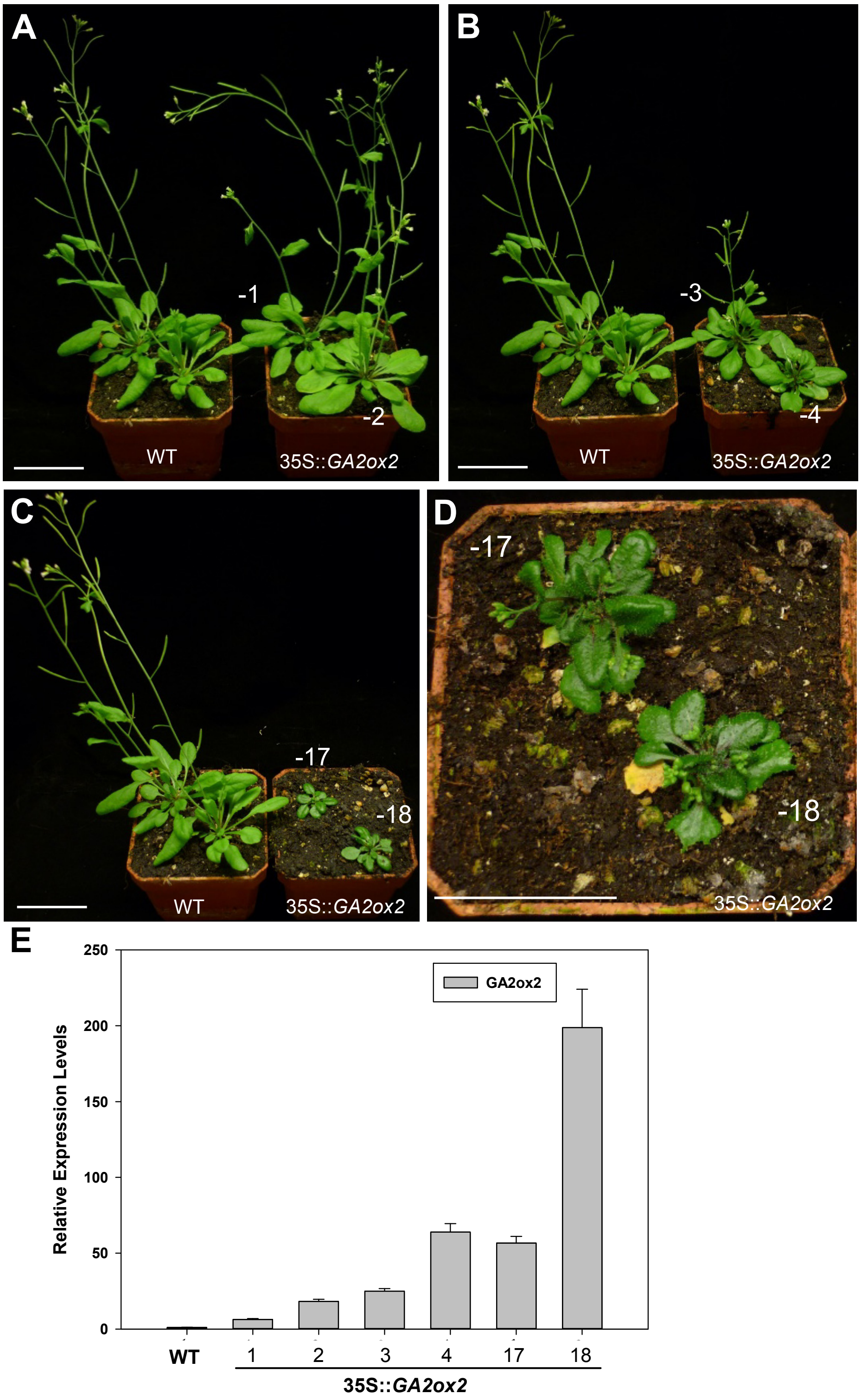

Supplementary Figure S4. Phenotypic analysis of the 35S::*GA2ox2 Arabidopsis* plants.
(A-C) 35S::*GA2ox2 Arabidopsis* plants showed either a weak **(A,** lines 1, 2), medium **(B,** lines 3, 4) or severe **(C,** lines 17, 18) dwarfism phenotype compared to the wild-type (WT) Arabidopsis.
(D) A close-up of two 35S::*GA2ox2 Arabidopsis* plants (lines 17, 18) showed severe dwarfism phenotype from **(C)**. Bar = 30 mm in (**A-D**).
(E) The detection of the expression for *GA2ox2* in six 35S::*GA2ox2* plants (lines 1, 2, 3, 4, 17 and 18) from **(A-C)** by real-time quantitative RT-PCR.

Chen_Fig.S5

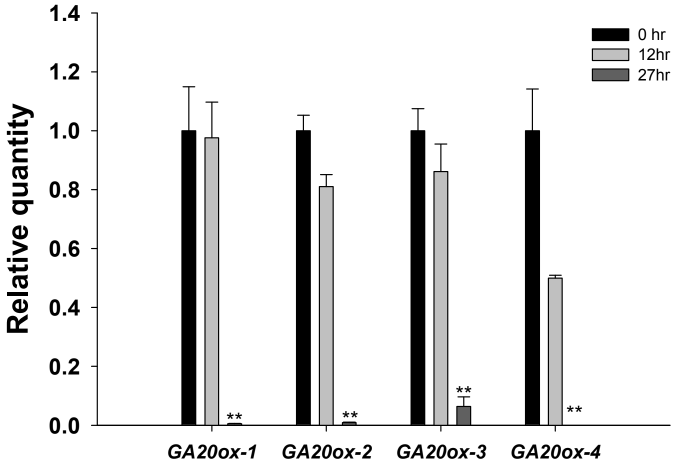

Supplementary Figure S5. Detection of the expression for *GA20oxs (1-4)* using real-time quantitative RT-PCR for 14-day-old wild-type *Arabidopsis* after exposure to 4ºC over a period of 12 and 27 hours. The expression of *GA20oxs (1-4)* was significantly down-regulated at 27 hours after cold treatment. The transcript levels of *GA20oxs (1-4)* were determined using three replicates and normalized against *UBQ10*. The expression level for each gene in untreated wild-type plants (time 0) was set at 1. Error bars represent the standard deviation. The asterisks indicate a significant difference from the untreated wild type plants (time 0) value (*means $P \leq 0.05$, **means $P \leq 0.01$). Statistic analysis was measured by student's T-test.

Chen_Fig.S6

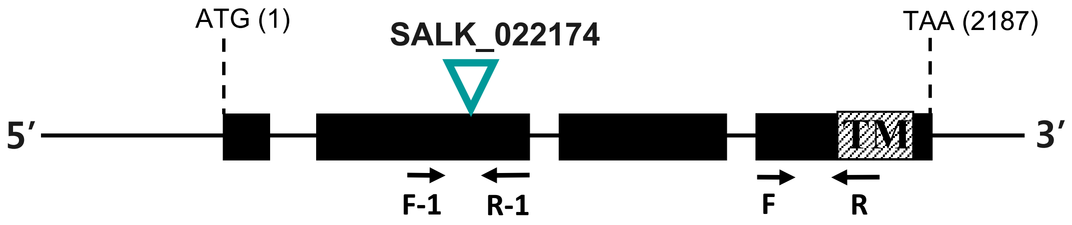

Supplementary Figure S6. Genomic region of *GSF*. Filled black boxes represent exons for *GSF* cDNA. Triangle indicates the location of T-DNA insertion in the second exon region in SALK_022174 mutants. For the detection of *GSF* expression in SALK_022174 mutant, the primers pair F-1 (GSF qRT for-1) and R-1 (GSF qRT rev-1), which located in the two sides of the T-DNA insertion, were used. For the detection of *GSF* expression in wild-type (WT) Arabidopsis, the primers pair F (GSF qRT for) and R (GSF qRT rev) were used.

Table S1 Oligo nucleotide sequence of primers used in gene cloning and PCR analysis.

Gene name	Primer name	Primer sequence	Restriction site	Useing
GSF	GSF-for	5'-GGTACCGATCGGTTTTGATTTGTTACGTAG-3'	*Kpn*I	Cloning
	GSF-rev	5'-GAGCTCGGTTTCTTCTTCTAACTTACCAGTAG-3'	*Sac*I	Cloning
	GSF-for-atg (*Hind*III)	5'-AAGCTTATGGCGGATTCTTCACCCGATTC-3'	*Hind*III	Cloning
	fuse-GSF-rev	5'-GAGCTCCTAGTCTTTCAAGAGAAGACTTC-3'	*Sac*I	Cloning
	GSF-TM-rev (*Sac*I)	5'- GAGCTCCTATCTGTTTCTAATACCCTTCTTTGCT-3'	*Sac*I	Cloning
	GSF qRT for	5'-AGATGAACTAGGGAGATGTAAGAACC-3'		Real time PCR
	GSF qRT rev	5'-GCATTAGGGATTCCATTGAGAAGC-3'		Real time PCR
	GSF qRT for-1	5'- AGATGAACTAGGGAGATGTAAGAACC-3'		Real time PCR
	GSF qRT rev-2	5'- GCATTAGGGATTCCATTGAGAAGC-3'		Real time PCR
	GSF-for-atg-Y	5'-CATATGGCGGATTCTTCACCCGATTC-3'	*Nde*I	Cloning
	GSF N-557 rev	5'-GTCGACCTAGTCTTTCAAGAGAAGACTTCT-3'	*Sal*I	Cloning
	GSF N-521 rev	5'-GTCGACCTAAATACCCTTCTTTGCTATGGT-3'	*Sal*I	Cloning
	GSF N-160 rev	5'-GTCGACCTAAAGAGCATAGTACTCCTGAGG-3'	*Sal*I	Cloning
	GSF N-161 for	5'-CATATGTATAAGTTGTTCAAGAAAAGTGGG-3'	*Nde*I	Cloning
	GSF-Pro-for	5'-CTGCAGCAAAACTCATCTGTTTCTCTTTTTG-3'	*Pst*I	Cloning
	GSF-Pro-rev	5'-GGATCCCTACGTAACAAATCAAAACCGATCC-3'	*Xba*I	Cloning
GFP-Gly-Ala	FmGFP5L-for (KpnI)	5'-GGTACCATGGTAGATCTGACTAGTAAAGGA-3'	*Kpn*I	Cloning
	FmGFP5L-rve I	5'-TCCAGCACCTGCTCCGCTAGCTTTGTATAGTTCATCCA-3'		Cloning
	FmGFP5L-rev II (HindIII)	5'-AAGCTTTGCTCCAGCACCTGCTCCAGCACCTGCTCCGCTA-3'	*Hind*III	Cloning
GAL4AD	GAL4 AD for	5'-CATATGGATAAAGCGGAATTAATTCCC-3'	*Nde*I	Cloning
	GAL4 AD rev	5'-GTCGACTTACTCTTTTTTTGGGTTTGG-3'	*Sal*I	Cloning
UBQ *At4g05320*	RT-UBQ10-F3	5'-CTCAGGCTCCGTGGTGGTATG-3'		Real time PCR
	RT-UBQ10-R3	5'-GTGATAGTTTTCCCAGTCAACGTC-3'		Real time PCR
GA2ox1 *At1g78440*	At1g78440 qRT for	5'- CAAGAGCGTGAGGCATAGGG-3'		Real time PCR
	At1g78440 qRT rev	5'- AGTCAATGAAGGTCCAGCGAAG-3'		Real time PCR
GA2ox2 *At1g30040*	AtGA2ox2 for PstI	5'-CTGCAGTAAAGATTTTGCAAGTTAAGTGT-3'	*Pst*I	Cloning
	AtGA2ox2 rev SalI	5'-GTCGACAAAGGATGATAAAGATCATCATG-3'	*Sal*I	Cloning
	At1g30040 qRT for	5'-CATTCTCTGCGGTTTGTTTGG-3'		Real time PCR
	At1g30040 qRT rev	5- CGTGAGTCTCAGTGTCTACATAG-3'		Real time PCR
GA2ox3 *At2g34555*	At2g34555 qRT for	5'-TGCCTGAGAATGAACCATTACCC-3'		Real time PCR
	At2g34555 qRT rev	5' TGTTCCATCTTTGACACAGATTTGC 3'		Real time PCR
GA2ox4 *At1g47990*	At1g47990 qRT for	5'-GCTCGGCAGTGAATTGTTACATAG-3'		Real time PCR
	At1g47990 qRT rev	5'-CACAGATTGGTCAGAAAGATTGGC-3'		Real time PCR
GA2ox6 *At1g02400*	At1g02400 qRT for	5'-ACAGAAGTCTAGCGAAGTGAGTG-3'		Real time PCR
	At1g02400 qRT rev	5'-CGGTGCTGGTGGATAGTGATTC-3'		Real time PCR
GA2ox7 *At1g50960*	At1g50960 qRT for	5'-TACCAAAGCGTGAGACATAGAGTG-3'		Real time PCR
	At1g50960 qRT rev	5'-CGAGATAAGGACATACGAAGAAAGC-3'		Real time PCR
GA2ox8	At4g21200 qRT for	5'-ATCCACCTTGTCCCAAACCATC-3'		Real time PCR

At4g21200	At4g21200 qRT rev	5'-AACAGCGATCCATCTATTGTCTTTG-3'		Real time PCR
GA20ox1 *At4g25420*	At4g25420 (GA20ox1)-for	5'- GGTACCCGCAATACTACTACTCACTTTACT-3'	*Kpn*I	Cloning
	At4g25420 (GA20ox1)-rev	5'- GAGCTCACTAGTAACTAACAAGACAAGACA-3'	*Sac*I	Cloning
	At4g25420 qRT for	5'-AGCCAATATCCCAAACCAATTCATC-3'		Real time PCR
	At4g25420 qRT rev	5'-TCGGAGAGAGGCATATCAAAGAAG-3'		Real time PCR
GA20ox2	At5g51810 qRT for	5'-CGGCAGAGAAAGAACACGAAC-3'		Real time PCR
At5g51810	At5g51810 qRT rev	5'-AGAGAAGGGTTAAAGATTAGTGGAG-3'		Real time PCR
GA20ox3	At5g07200 qRT for	5'-GCCTCTTGTCTCGTGCCTATC-3'		Real time PCR
At5g07200	At5g07200 qRT rev	5'-CACTTCCTCTGAGCCTTCTGC-3'		Real time PCR
GA20ox4	At1g60980 qRT for	5'-TCAACCAGACCATATACCTCAAGAG-3'		Real time PCR
At1g60980	At1g60980 qRT rev	5'-TTCAGCCTCCGAGACCAATAATG-3'		Real time PCR
